# Carbon dot-protoporphyrin IX conjugates for improved drug delivery and bioimaging

**DOI:** 10.1371/journal.pone.0220210

**Published:** 2019-07-25

**Authors:** Jose R. Aguilar Cosme, Helen E. Bryant, Frederik Claeyssens

**Affiliations:** 1 Department of Materials Science and Engineering, Kroto Research Institute, University of Sheffield, Sheffield, United Kingdom; 2 Department of Oncology & Metabolism, The Medical School, University of Sheffield, Sheffield, United Kingdom; Massachusetts General Hospital, UNITED STATES

## Abstract

Photodynamic therapy (PDT) uses photosensitisers such as protoporphyrin IX (PpIX) to target tumours via the release of toxic singlet oxygen when irradiated. The effectivity of the treatment is limited by the innate properties of the photosensitizers; they typically exhibit inefficient accumulation in target tissue and high dark toxicity. Carbon dots (CDs) are biocompatible fluorescent nanoparticles which can improve PpIX cellular uptake and solubility. In this work, we present conjugates synthesised by host-guest encapsulation (PpIX@CD) and amide cross-linking (PpIX-CD). Characterization demonstrated conjugates have a loading efficiency of 34–48% and similar singlet oxygen production to PpIX. PpIX-containing CDs showed a 2.2 to 3.7-fold decrease in dark toxicity. PpIX-CD and PpIX@CD showed equivalent light-induced toxicity to PpIX in concentrations >1 μg/ml, leading to a 3.2 to 4.1-fold increase in photo-toxicity index (PI). The less soluble fraction of cross-linked conjugates (PpIX-CD)p did not show significant difference from PpIX. Confocal light scanning microscopy demonstrated rapid intracellular uptake and accumulation of conjugates. Our results demonstrate the variations between cross-linking strategies in CD-based conjugates, highlighting their potential as carriers in drug delivery and bioimaging applications.

## Introduction

Photodynamic therapy (PDT) has seen advances in recent years as an alternative cancer treatment due to its non-invasive nature, specificity and selectivity [1]. The term “PDT” describes a range of protocols based on the excitation of photosensitizers (PS) in the presence of oxygen to singlet oxygen (^1^O_2_) leading to tumour ablation [2]. PDT has been proven to be clinically effective presenting positive results in basal cell carcinoma, endobronchial lung cancer, and non-muscle invasive bladder cancer [3–5]. Highly controlled light dosimetry and rapid drug uptake maximizes the effectiveness of the treatment and prevents damage to surrounding tissue [6].

Nanoparticle-photosensitizer conjugates have received increased interest due to several advantages such as; (i) large surface-volume ratios for increased loading efficiency, (ii) the formation of amphiphilic compounds to avoid aggregation, and (iii) the enhanced permeability and retention effect for increased accumulation in tumours due to “leaky” vasculature [7–9]. Moreover, conjugates can also function as bioimaging probes to form multifunctional theragnostics platforms through photodynamic diagnosis (PDD) [10]. Porphyrins are naturally-occurring heterocyclic molecules composed of pyrrole rings connected by methylylidene bonds. These molecules are found in living organisms acting as electron and oxygen transporters or metalloenzymes through the chelation of metal ions by coordination.

Protoporphyrin IX (PpIX) is a well-characterised endogenous porphyrin photosensitizer, normally present in minor concentrations within cells as part of the heme biosynthesis pathway. Dormant cancer cells have been proven to accumulate high concentrations of PpIX and are more susceptible to PDT [11]. However, PpIX is limited as a photosensitizer mainly due to elevated dark toxicity and rapid aggregation. This which leads to decreased photoactivity as singlet oxygen production is attenuated [12,13]. Recent advances have focused on utilising carriers and chemical modifications to improve water solubility and increase cellular viability [14]. For example, Homayani *et al*. (2015) demonstrated that hydroxyl-group modification can increase the water solubility of PpIX, reducing dark toxicity and increasing cellular uptake [15].

Carbon dots (CDs) are carbon-based fluorescent nanoparticles that have gained attention due to their interesting photophysical properties, low toxicity, tuneable surface functionality and adaptable synthesis making them ideal candidates for drug delivery, bioimaging, and theranostics applications. [16–19]. CDs have shown similar success in biomedical applications in comparison to other nanomaterials such as semiconductor quantum dots, nanodiamonds, graphene, and carbon nanotubes. Nanoparticle-based drug delivery has been shown to improve intracellular drug uptake and reduce the likelihood of cargo degradation. [20]. The rapid intracellular uptake of CDs and CD-based conjugates has been shown to be time and dose-dependent and is a combination of both passive uptake and caveolae and clathrin-mediated endocytosis [21]. Moreover, CDs can be further modified by doping with heteroatoms and surface passivation with a variety of molecules such as polyethylene glycol to achieve better photophysical properties [22]. CDs have previously been used as carriers for a wide variety of compounds, including doxorubicin, rhodamine B, dsDNA, siRNA and ciprofloxacin hydrochloride [23–27].

CDs have tuneable photoluminescence ranging across the visible spectrum which depends on their synthesis conditions, affecting quantum yield, determining excitation-dependent or independent emission, and type of photoluminescence decay [28]. *In vitro* studies demonstrate rapid intracellular uptake and do not show significant toxicity even at extremely high concentrations [29]. *In vivo* and *ex vivo* imaging in BALB/c mice show similar results with no observable toxicity and rapid clearance from the reticuloendothelial system [30]. Furthermore, CDs have previously demonstrated comparable two-photon cross-sections to those of commercially-available quantum dots, making them highly valuable as probes for bioimaging applications [31].

Recently, CD-PS cross-linking has recently gained research interest. CDs have extremely high surface area to volume ratios which make them ideal candidates for drug loading and cross-linking. Amide cross-linking is a well-known cross-linking strategy which relies on interactions between carboxyl and primary amine groups. EDC (1-ethyl-3-(3-dimethylamino) propyl carbodiimide) is a zero-length cross-linker that can be used alongside *N*-hydroxysuccinimide (NHS) to efficiently produce amide bonds between two suitable molecules [32]. Photosensitizers such as chlorin e6, Rose Bengal and PpIX have been previously covalently linked through carbodiimide chemistry, the latter of which showed a PDT effect under two-photon excitation [33–35]. Similarly, recent advances have shown embedded photosensitizers are capable of singlet oxygen production while embedded on nanoparticles [36,37]. Host-guest embedding reduces the complexity and costs of synthesis while retaining drug loading.

In this study, we present PpIX@CD, a CD-based conjugate synthesized through a one-step microwave synthesis route. PpIX-CD was synthesized through an adapted carbodiimide cross-linking protocol. The less soluble fraction (PpIX-CD)p was recovered during sample purification of cross-linked conjugates and evaluated against PpIX-CD and PpIX@CD. Singlet oxygen production was observed for all samples with phenalenone as a standard. The aim of this study is to compare the photophysical properties and *in vitro* toxicity of PpIX-CD, (PpIX-CD)p and PpIX@CD to PpIX in human melanoma (C8161) monolayer cell cultures and determine the optimal drug loading strategy for increasing PDT efficiency. Conjugates were also evaluated as bioimaging probes in human osteosarcoma (U2-OS) using confocal laser scanning microscopy.

## Experimental

### Materials

Citric acid monohydrate, sucrose, ethylenediamine, protoporphyrin IX, sodium chloride, resazurin sodium salt, (N-(3-Dimethylaminopropyl)-N’-ethylcarbodiimide), N-Hydroxysuccinimide, formaldehyde, phenalenone, 2-(N-Morpholino) ethanesulfonic acid, acetone, dimethyl sulfoxide, 2-mercaptoethanol and N,N-dimethylformamide were acquired from Sigma Aldrich (United Kingdom). Dulbecco’s Modified Eagle’s Medium (DMEM, high glucose), Dulbecco’s Modified Eagle’s Medium (DMEM, high glucose, without phenol red), fetal bovine serum (FBS), phosphate buffer saline (PBS), and trypsin–ethylenediaminetetraacetic acid solution were obtained from Thermo Fisher (United Kingdom). Syringe filters with a 0.2 μm pore size were acquired from Sarstedt (United Kingdom). 1 KDa MWCO, 6.4 ml/cm dialysis tubing was acquired from Spectrum Labs (United States of America). All chemicals were used as received unless stated otherwise. Deionized water was used for all buffers and samples in experiments. Septa steel ring caps and 35 ml glass reaction vessels were obtained from CEM Corporation (United Kingdom).

### Sample preparation

CA-EDA CDs were synthesized utilising 5 g of citric acid and 1.25 g of EDA dissolved in 100 ml of deionized water and stirred until no visible precipitate remained. This process was repeated for S-EDA CDs with 5 g of sucrose and 1.25 g EDA. A separate solution was used in host-guest embedding (PpIX@CD), with 0.5 mg/ml protoporphyrin IX, 5 g of citric acid and 1.25 g EDA in deionized water. A CEM Discover SP microwave reactor was used to heat the precursor solutions for 5 minutes at constant 150°C (200 W maximum power and 17 bar threshold). The resulting yellow-coloured solution was cooled to room temperature and centrifuged to remove debris at 5000 rotations per minute (rpm) for 30 minutes. Samples were dialysed against deionised water with a 1 KDa MWCO membrane for 48 hours, changing the buffer at six-hour intervals. Freeze drying was used to remove all liquids from the solutions (-10°C shelf temperature, -70°C collector, 48 hours, 0.1 mbar). The remaining powder was weighed and stored for further use at 4°C.

### Amide cross-linking of CDs and PpIX

A 4 mg/ml PpIX solution was prepared by dissolving the powder in 25 ml N,N-dimethylformamide and stirred for 30 minutes at room temperature, keeping it in the dark. Afterwards, 25 mg (N-(3-Dimethylaminopropyl)-N’-ethylcarbodiimide) and 50 mg N-Hydroxysuccinimide were added and kept stirring for an additional 30 minutes until no precipitate could be seen. Subsequently, 100 mg of CDs were dissolved in 25 ml PBS and added to the previous solution. The solution was stirred overnight and centrifuged, separating the less soluble precipitate (PpIX-CD)p. The solutions were dialysed against deionised water using a 1 KDa MWCO membrane for 48 hours, changing the buffer at six-hour intervals or when a visible colour change could be observed. Finally, samples were freeze-dried and stored at 4°C. A stock solution of conjugates was prepared at a concentration of 1 mg/ml utilising deionised water. The stock solutions were kept frozen at—20°C until used.

### Conjugate characterization

Transmission electron microscopy (TEM) was used to obtain high-magnification images utilising 1 μg/ml solutions of conjugates that were previously processed with the ultrasonic probe. Samples were observed on carbon coated grids by a FEI Tecnai G2 Spirit TEM. Individual particles were counted and measured to determine average particle size. Ultraviolet-visible spectroscopy (UV-Vis) was carried out utilising a PerkinElmer Lambda 900 spectrometer in the range of 250 to 750 nm. Fluorescence spectra were recorded with a Fluoromax 4 fluorometer utilising 405 nm excitation wavelength, 0.5 nm path length, and 0.5 nm increments from 415 to 750 nm. PpIX loading was calculated by fluorescence comparisons as reported previously [38]. A sample concentration of 1 μg/ml conjugate in deionised water was utilized for both spectroscopies. Fourier-transform infrared spectroscopy (FTIR) was used to obtain spectra with a Nicolet iS50R FT-IR in photoacoustic mode; samples were scanned 512 times in the range of 450 to 3500 cm^-1^. X-ray photoelectron spectroscopy (XPS) analysis was carried out with a monochromated Al-kα X-ray source, two analysis points per sample and a total scan area of 700 x 300 μm using an Axis Ultra DLD system (Kratos Analytical, United Kingdom). 5 mg of conjugate powder samples were mounted on between indium foil and a paper label to mitigate the risk of differential charging. Survey scans were collected in the range of 1200 to 0 eV binding energy (160 eV pass energy, 1 eV intervals, and 300 seconds per sweep–with 4 sweeps collected). High-resolution C1s spectra were collected at 20 eV pass energy and 0.1 eV intervals. The influence of indium foil on each sample was removed taking into account a surface composition of 26.8 at% O, 19.4 at% In, and 53.8 at% C.

### Singlet oxygen production

PpIX, PpIX-CD, (PpIX-CD)p, PpIX@CD, and PNP were dissolved in DMF and the solution absorbance was adjusted to 0.1–0.12. The solutions were covered in aluminium foil to prevent photobleaching. A Nd: YAG 355 nm laser was used to excite the solutions at 50, 100 and 200 mJ. Phenalenone was utilized as a control to indicate 100% production. The characteristic fluorescence of singlet oxygen (in the range of 1160–1380 nm) was resolved and collected with an infrared-sensitive photomultiplier as previously reported [39].

### Evaluation of cytotoxicity

Nanoparticle-supplemented DMEM was prepared utilising a stock solution of each conjugate, at concentrations from 1–100 μg/ml. All solutions were subjected to ultrasonic processing with a Hieschler UP50H ultrasonic probe and filter sterilised prior to use in cell culture. Growth media was prepared utilising phenol red-free DMEM with the following: 10% fetal calf serum, 1% antibiotics (penicillin and streptomycin) and 1% glutamine. C8161 melanoma cells were used from passage 10 to 20 and were cultured in a T75 plate 5% CO_2_ at 37°C, until reaching approximately 90% confluence. Afterwards, cells were diluted to 6 × 10^4^ cells/ml; each well of a 96-well plate was seeded with 100 μl of cell suspension and placed in the incubator overnight to allow attachment.

#### Dark toxicity

Growth medium was replaced with 100 μl of conjugate dilutions (1–100 μg/ml) and DMEM was added to untreated cells to act as a control. The plates were covered and returned to the incubator for an additional 24 hours. After incubation, each well was washed with PBS and 200 μl growth media was replaced prior to the metabolic activity assay.

#### Light-activated toxicity

Growth medium was replaced with 100 μl of conjugate dilutions (1–10 μg/ml) and DMEM was added to untreated cells to act as a control. Cells were returned to the incubator for 3 hours to allow particle internalization. Afterwards, all wells were washed using PBS and 200 μl phenol red-free media was added. A M405L2 ThorLabs mounted LED with a collimator adapter (405 nm, 2.76 mW/cm^2^) was used to induce light-activated toxicity. Cells were placed under illumination for 3 minutes and subsequently returned to the incubator. Metabolic activity measurements were taken at 24, 48 and 72-hour time points (post light activation).

#### Metabolic activity assay

A 1 mM resazurin solution was prepared by dissolving 25.18 mg of resazurin sodium salt in 100 ml sterile PBS. The solution was filter sterilized using a 0.2 μm syringe filter. 100 μl of media was taken from each well and transferred to a new plate. Metabolic activity was assessed by adding 20 μl to each well. The plates were read using a Biotek ELx808 Microplate Reader at 570/585 nm with a sensitivity of 50. Conjugate LD_50_ values were obtained and converted into PpIX-adjusted concentrations (μM) based on the previous estimated PpIX content of each sample.

### Confocal light scanning microscopy

#### Preparation of samples

Fluorescence images were obtained using a Zeiss LSM510 Meta confocal microscope fitted with a two-photon Ti-Sapphire laser. U2-OS cells were seeded in six-well tissue culture plates at a density of 1 × 10^5^ cells per well. Cells were placed in the incubator for 2 hours to allow cell attachment. A 1 μg/ml solution of each conjugate was prepared. Wells were washed with PBS and growth media was replaced with 2 ml of conjugate solution. The plate was returned to the incubator for 30 minutes. Immediately afterwards the wells were washed with PBS and fixed using 3.7% formaldehyde and 300 nM 4′,6-diamidino-2-phenylindole.

#### Image acquisition

Images were obtained using 488 nm (15%), 543 nm (15%) and 800 nm (6.5%) laser lines. Confocal light scanning microscopy was performed using an Achroplan 40×/0.75 N.A. water immersion objective. Z-stacks were defined as a 210.4 ×210.4 × 7.2 μm area with a pixel time of 51.2 μs.

### Statistical analysis

Experiments carried out with three independent repeats in triplicates (N = 3, n = 3) and results were normalized using untreated controls. Statistical analysis was carried out using GraphPad Prism version 7.04. The comparison of metabolic activity was evaluated by 2-way ANOVA analysis with Dunnett’s test for multiple comparisons *P* values < 0.05 were considered statistically significant. Data was presented as means ± SEM (standard error of the mean).

## Results

### Characterization of PpIX@CD and PpIX-CD

**Fig 1:**
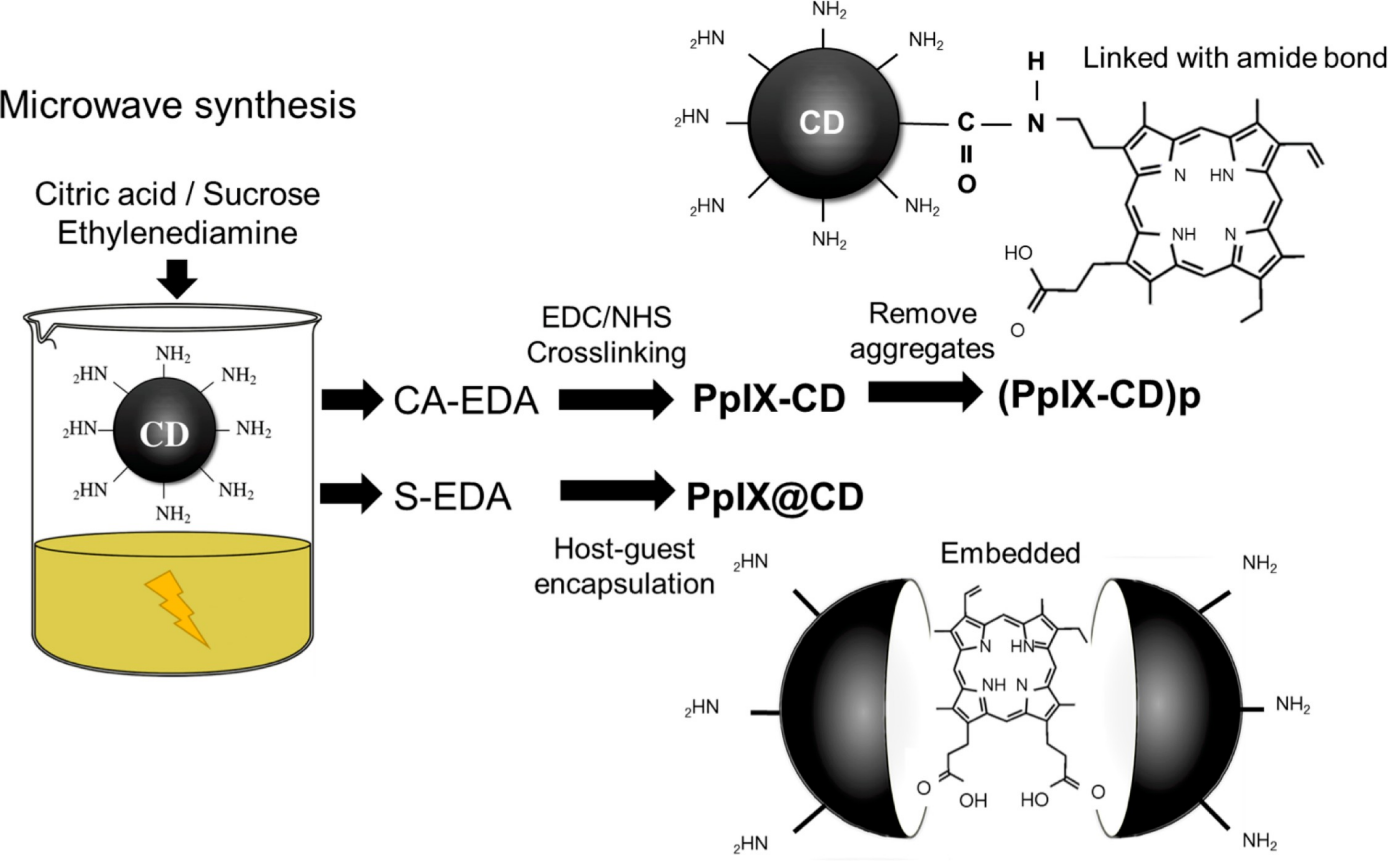
Synthesis routes utilized for CD-PS conjugates. Host-guest encapsulated (PPIX@CD) samples were produced in a one-pot reaction. CA-EDA CDs were used to produce amide bond-linked (PPIX-CD and (PpIX-CD)p) conjugates. S-EDA CDs were embedded with PpIX in a one-pot encapsulation step.

CDs were synthesized utilizing microwave-assisted pyrolysis of a carbon source (citric acid or sucrose), EDA functioning as both passivating agent and amine source. (Fig 1). The use of ethylenediamine in CA-EDA and S-EDA CDs ensures the availability of primary amine functional groups which can be used in cross-linking. The optimal temperature and time were determined to be 150°C and 5 minutes respectively to prevent excessive formation of aggregates. This methodology was utilized to produce CA-EDA (citric acid-based) and S-EDA (sucrose-based) CDs.

Conjugates were synthesized with two distinct strategies (Fig 1). PpIX-CD was fabricated with a modified cross-linking protocol which formed a covalent amide bond between the amine groups of CA-EDA CDs and carboxylic acid groups of PPIX. PpIX@CD was successfully synthesized by a one-pot reaction of sucrose, EDA and PpIX, and was recovered as a dark red liquid. An ultrasonic probe was used to thoroughly mix each solution prior to pyrolysis.

Purification was carried out in several steps throughout synthesis and cross-linking. We recovered the less soluble fraction of the amide cross-linking solution, named (PpIX-CD)p, with an increase of centrifugation time and speed. Dialysis with a 1 kDa MWCO membrane removed remaining contaminants from the CD and conjugate solutions. Freeze-drying produced a reddish to black powder which was subsequently weighed and stored in a dry environment away from light until used.

**Fig 2:**
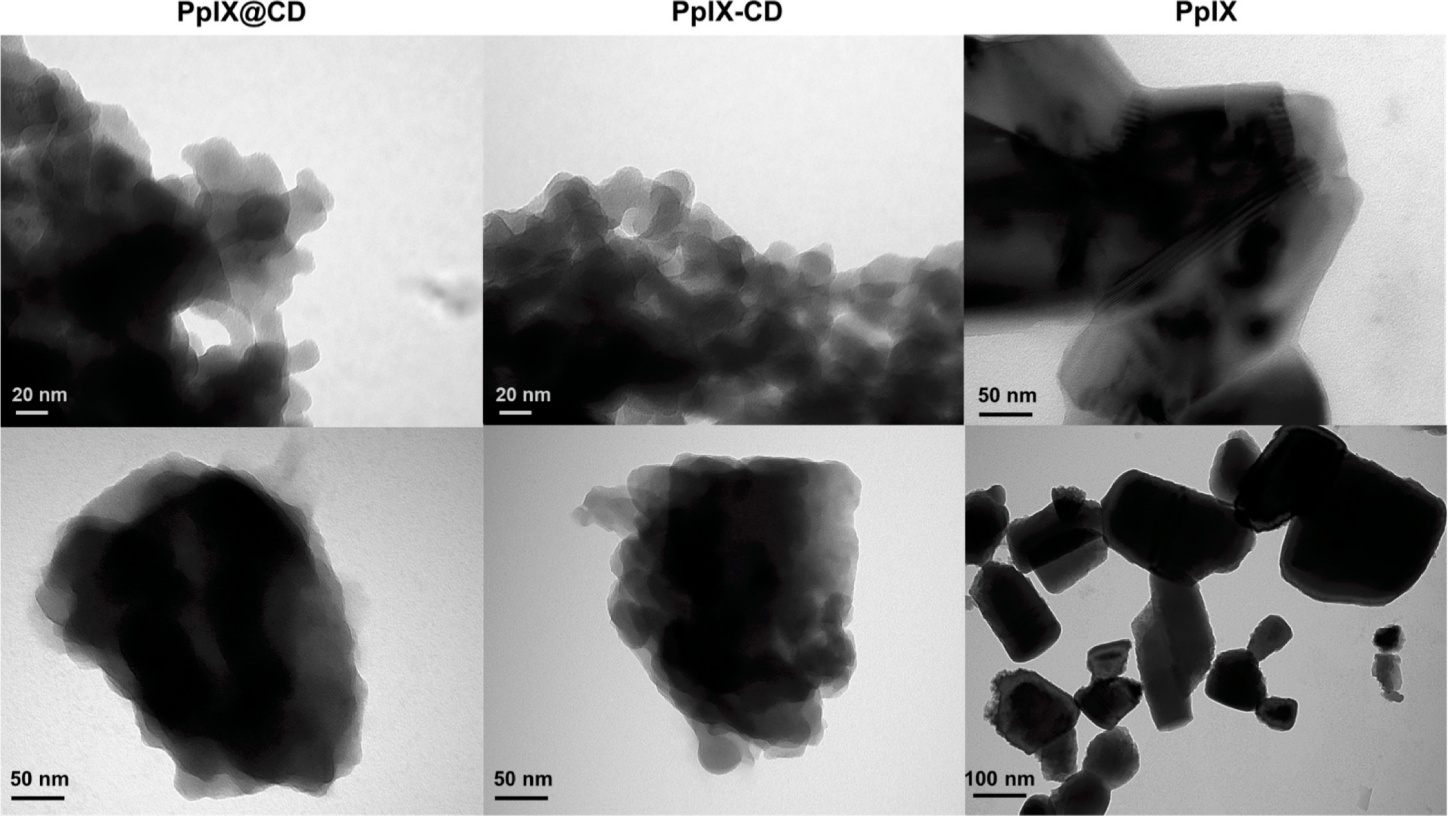
CD-PS conjugates show decreased aggregation in water. TEM images of conjugates at 30,000× (top) and 68,000× (bottom). Conjugates show irregular morphology and less aggregation in comparison to PpIX (30,000× top and 18,500× bottom).

Transmission electron microscopy (TEM) revealed a quasispherical particle morphology for all samples with an average diameter of 25 ± 10 nm (PpIX-CD) and 17 ± 6 nm (PpIX@CD) (Fig 2). (PpIX-CD)p exhibited a highly variable particle size range of 15–100 nm. (S1 Fig). Conjugates displayed an irregular quasispherical morphology, with aggregates forming regardless of the concentration and sample grid-loading combination that was tested. CA-EDA and S-EDA CDs showed an average particle size below 10 nm and a more defined spherical morphology (S2 Fig). Finally, PpIX coalesced into well-defined geometric structures with sizes greater than 100 nm in diameter. Our results show PpIX-CD and PpIX@CD form reduced aggregates under 200 nm in size while both (PpIX-CD)p and PpIX quickly form aggregates. Conjugates show reduced solubility in water in comparison to base CDs but are more soluble than PpIX in concentrations below 25 μg/ml. Solutions with conjugates show slight precipitation after several hours of ultrasonic processing.

**Fig 3:**
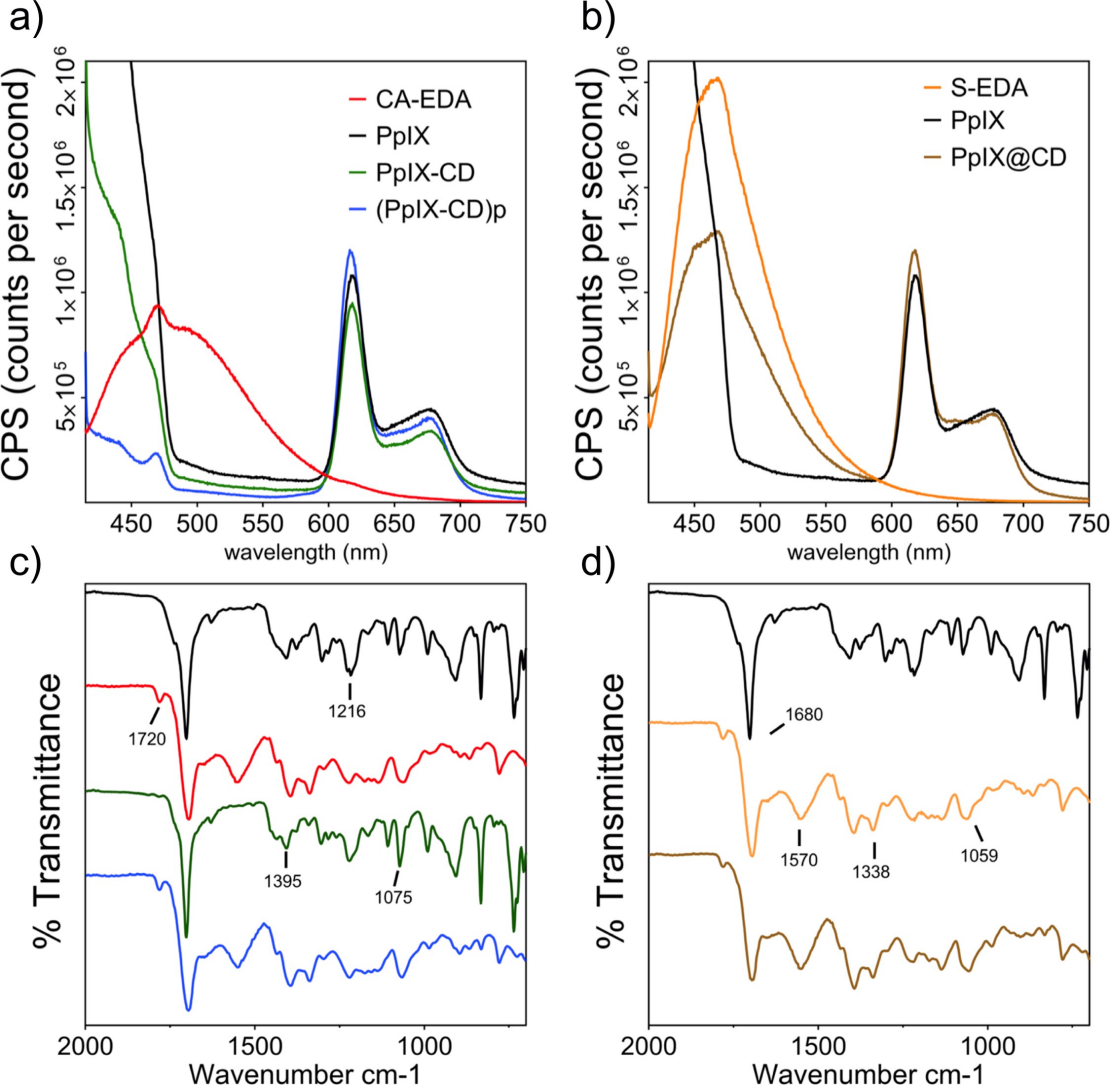
Drug loading reduces CD-based photoluminescence and affects surface chemistry. CD-PpIX conjugates demonstrate dual emission at λ_ex_ = 405 nm, with quenching of CD-based photoluminescence (a,b). FT-IR demonstrated particles could be separated into two groups based on their similarities to PpIX and CD spectra (c,d). The effect of amide cross-linking can be seen with the varying intensity of the amine band at 1570 cm^-1^.

We characterized the conjugates to determine similarities to PpIX. Fluorescence spectra were obtained utilising conjugates suspended in PBS at pH 7 (Fig 3B) by matching the maximum excitation wavelength to the LED used for *in vitro* tests (λ_ex_ = 405 nm). PpIX@CD was compared to S-EDA CDs while PpIX@CD and (PpIX-CD)p were compared to CA-EDA CDs. Fluorescence spectra demonstrate a dual emission behaviour from all conjugates. (Fig 3A and 3B) Carbon dot-related emissions are attenuated in conjugates, while PpIX-related emission peaks >600 nm are very similar between all samples. (PpIX-CD)p showed greatly reduced fluorescence to all samples in the range of 420–550 nm. PpIX loading in conjugates showed various ratios: PpIX-CD (41%), (PpIX-CD)p (34%), and PpIX@CD (48%).

Infrared spectra obtained from PpIX and PpIX-CD were found to be nearly identical. Similarly, this was observed between CA-EDA, S-EDA CDs, (PpIX-CD)p and PpIX@CD. FT-IR spectroscopy was used to evaluate conjugate surface chemistry in the range of 4000–700 cm^-1^ (Fig 3C, 3D and S3 Fig). CA-EDA CDs were found to be very similar to S-EDA CDs, possibly due to the similarity of their carbon sources. Peaks attributed to C-C stretching at 1680cm^-1^ can be seen in all samples. However, the small 1720 cm^-1^ peak corresponding to C = O stretching and the broad -OH peak at around 3000 cm^-1^ were not observed in PpIX-CD and PpIX. In comparison, these peaks were seen in all other samples including PpIX@CD and (PpIX-CD)p. The characteristic amide band can be observed in CD, (PpIX-CD)p and PpIX@CD around 1570 cm^-1^ and is absent in PpIX-CD. Peaks in the range of 1395–1216 cm^1^ can be ascribed to C = C, C = N, and C = C-O respectively. The small sharp peaks at 1075 and 1059 cm^-1^ can be seen in samples corresponding to C-O and C-H groups.

XPS analysis with survey and high resolution C1s scans determined conjugates have little to no difference in surface composition with PpIX. Carbon dot compositions are very similar between CA-EDA and S-EDA samples, with only slight variations in C and O. The pure PpIX sample is close to what is expected given its chemical formula (C_34_H_34_N_4_O_4_). If hydrogens in this sample are neglected, it has concentration values of 82.6 at% C, 8.2 at% N and 9.0 at% O, as seen in Table 1. In comparison, CD conjugates demonstrate small increases in oxygen. The high resolution C1s spectra peak positions are given relative to C-C/C-H being at ~285.0 eV, and it is assumed the lowest carbon peak position is C-C/C-H as no carbides were expected in these samples. (S1 Table) There was no distinction in peak position between both C-C and C = C type bonds and are therefore expected to be the major peak.

**Table 1:** Surface composition (atomic%) of PpIX and CD-conjugates.

Sample	C	O	N	Na	Cl
CA-EDA	56.7	37.4	5.8	<0.1	0.1
S-EDA	59.1	35.4	5.4	<0.1	0.1
PpIX	82.6	9.0	8.2	<0.1	0.2
PpIX-CD	81.6	9.6	8.6	<0.1	0.2
(PpIX-CD)p	76.4	13.5	9.7	<0.1	0.4
PpIX@CD	82.1	9.4	8.3	<0.1	0.2

**Fig 4:**
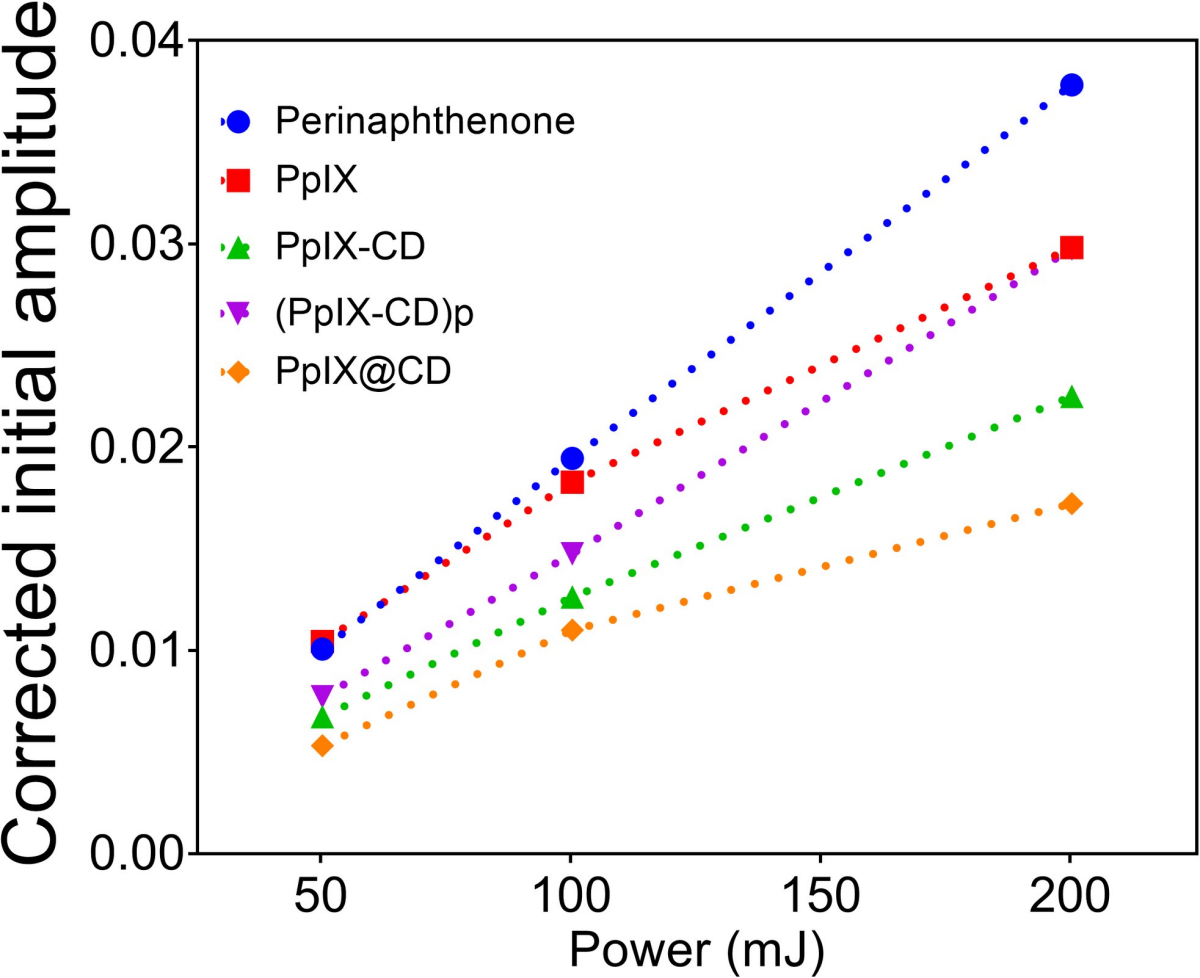
Singlet oxygen yield of conjugates in DMF. Corrected initial amplitude of lifetime generated singlet oxygen against the power of a 355 nm Nd:YAG laser to calculate singlet oxygen yield of each sample. Phenalenone was used as a control for 95% production.

Singlet oxygen production was determined by the time-resolved measurement of its characteristic luminescence at 1270 nm (Fig 4). Samples were excited utilising a 355 nm Nd:YAG laser at 50, 100, and 200 mJ. Phenalenone (PH) in dimethylformamide (DMF) was used as a standard indicating 100% singlet oxygen production. PpIX was determined to produce an average of 92.18% singlet oxygen. ^1^O_2_ production in conjugates was also calculated with values of 63.79% (PpIX-CD), 77.10% (PpIX-CD)p and 51.62% PpIX@CD.

### Evaluation of dark and light-activated toxicity

We evaluated the two parameters for determining conjugate cytotoxicity–dark toxicity (inherent toxicity of the particles prior to light exposure) and light-activated toxicity. The average lethal concentration at which metabolic activity is reduced by 50% (LD_50_) was estimated using these concentrations. We also calculated the photo-toxicity index (PI) to make direct comparisons between conjugates and PpIX. The PI index links dark and light-activated toxicity–higher PI values indicate greater efficiency with lower photoactivation LD_50_ concentrations and increased dark toxicity resistance to PS. Cellular uptake and toxicity were evaluated in human melanoma (C8161) cells using the Resazurin reduction assay to estimate metabolic activity. DMEM-conjugate solutions over 10 μg/ml exhibited a distinct colour change from golden yellow to increasingly darker shades of red with the addition of both PpIX and conjugates.

**Fig 5:**
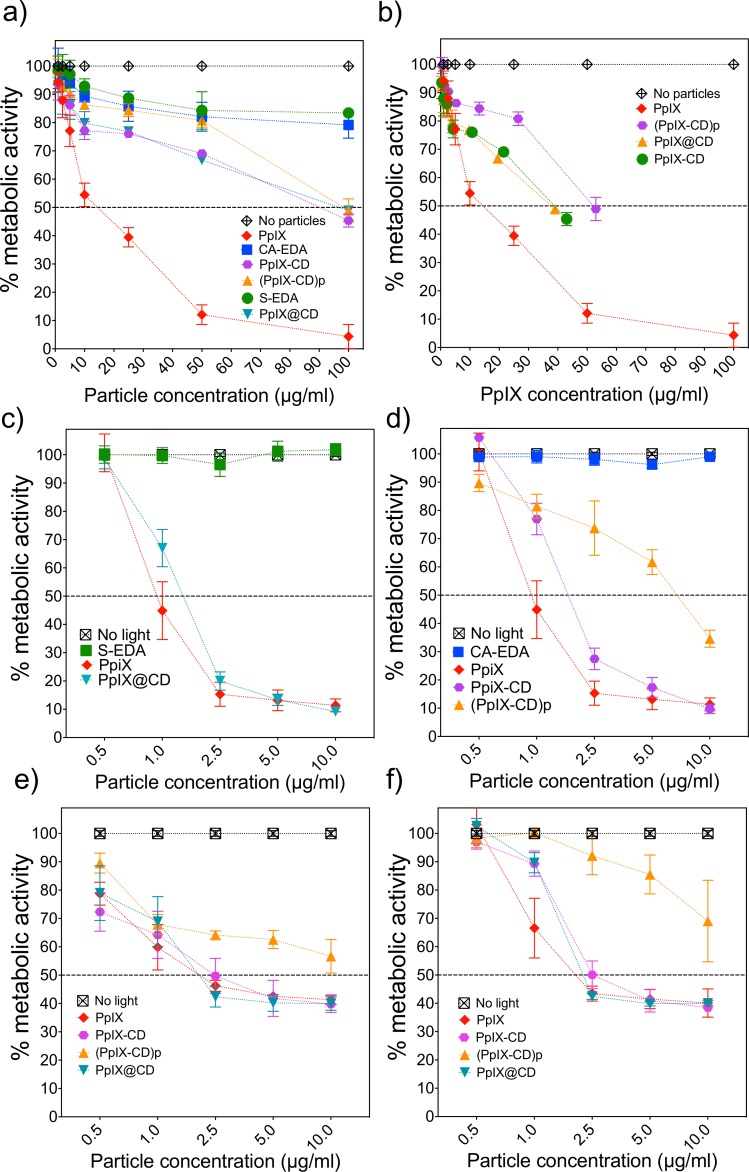
PpIX-CD and PpIX@CD decrease dark toxicity and produce an equivalent phototoxic effect to PpIX. Metabolic activity was measured after 24 hours post-conjugate incubation. Dark toxicity was shown as particle concentration (a) and total PpIX content (b). Light-activated toxicity was measured after a 3-minute time exposure in embedded (c) and cross-linked (d) conjugates (λ_ex_ = 405 nm, 2.78 mW/cm^2^). Further evaluation at 48 (e) and 72 (f) hours show a recovery of metabolic activity in all samples.

Dark toxicity was reduced in all conjugates when the porphyrin is bound to the CDs. CA-EDA and S-EDA CDs were used as controls for CD-induced toxicity and showed high biocompatibility at concentrations over 100 μg/ml. Fig 5A shows a gradual decrease in metabolic activity in proportion with conjugate concentration. We observed all conjugates exhibited LD_50_ values (μg/ml) approximately 6-fold greater than free PpIX, with adjusted LD_50_ values over 3-fold higher than the drug alone. PpIX@CD exhibited the highest LD_50_ of all samples while containing the highest PpIX concentration at 80.8 μM PpIX-adjusted (95.4 μg/ml), compared to PpIX-CD (64 μM PpIX-adjusted, 88.5 μg/ml) and (PpIX-CD)p (60.3 μM PpIX-adjusted, 100.5 μg/ml).

Light activated toxicity of conjugates demonstrated similar LD_50_ values between PpIX, PpIX-CD and PpIX@CD (Fig 5C–5F). (PpIX-CD)p showed a significant difference from all other samples with a reduced PDT effect at concentrations higher than 2.5 μg/ml. Incubation with free PpIX induced significant light induced toxicity with an LD_50_ of 1.68 μM. Table 2 shows these values are similar to the PpIX-adjusted concentrations for PpIX-CD and PpIX@CD (LD_50_ 1.4 μM and 1.3 μM respectively). The photo-index (PI) of compounds was calculated as the concentrations for dark toxicity divided by light toxicity. PpIX (μM) -CD and PpIX@CD PI values were improved significantly from 14.6 to 46.6 and 59.6 respectively compared to free PpIX.

**Table 2:** PpIX-CD conjugates improve PDT efficiency.

PpIX (%)	Singlet Oxygen		Dark		Light		PI
	Sample	Average ^1^O_2_ Yield	LD_50_ (μg/ml)	PpIX (μM)	LD_50_ (μg/ml)	PpIX (μM)	
0	CA-EDA	0	>100	N/A	0	0	N/A
0	S-EDA	0	>100	N/A	0	0	N/A
100	PpIX	92.2	14.6	25.8	1.0	1.7	14.6
41	PpIX-CD	63.8	88.5	67.3	1.9	1.4	46.6
34	(PpIX-CD)p	77.1	100.5	94.2	7.2	4.3	13.9
48	PpIX@CD	51.6	95.4	59.9	1.6	1.3	59.6

Comparison of singlet oxygen yield, dark toxicity and light-activated efficiency of samples versus PpIX with measured and PPIX-adjusted values.

Cell cultures were monitored for an additional (Fig 5E) 48 and (Fig 5F) 72 hours post light exposure. Metabolic activity *in vitro* shows recovery at 48 and 72 hours post light exposure regardless of conjugate type or concentration. We observed PpIX-CD and PpIX@CD followed the same pattern as PpIX >1 μg/ml at all time points. However, (PpIX-CD)p continuously exhibited decreased phototoxicity at all concentrations with high variability.

PpIX-CD and PpIX@CD exhibited increased PI values in comparison to PpIX, with a 2.8 and 3.5-fold increase. Both conjugates showed significant difference from PpIX ≤1 μg/ml (24 hours) but did not show a significant difference at concentrations of >2.5 μg/ml in any time point. We observed PDT efficiency was improved due to the decrease of dark toxicity of conjugates. In comparison, (PpIX-CD)p was found to have reduced PDT effect primarily due to its low phototoxic effectiveness. Furthermore, this sample consistently showed a statistically significant difference from light-activated PpIX at concentrations above 1 μg/ml.

### Bioimaging of CD-PS conjugates

**Fig 6:**
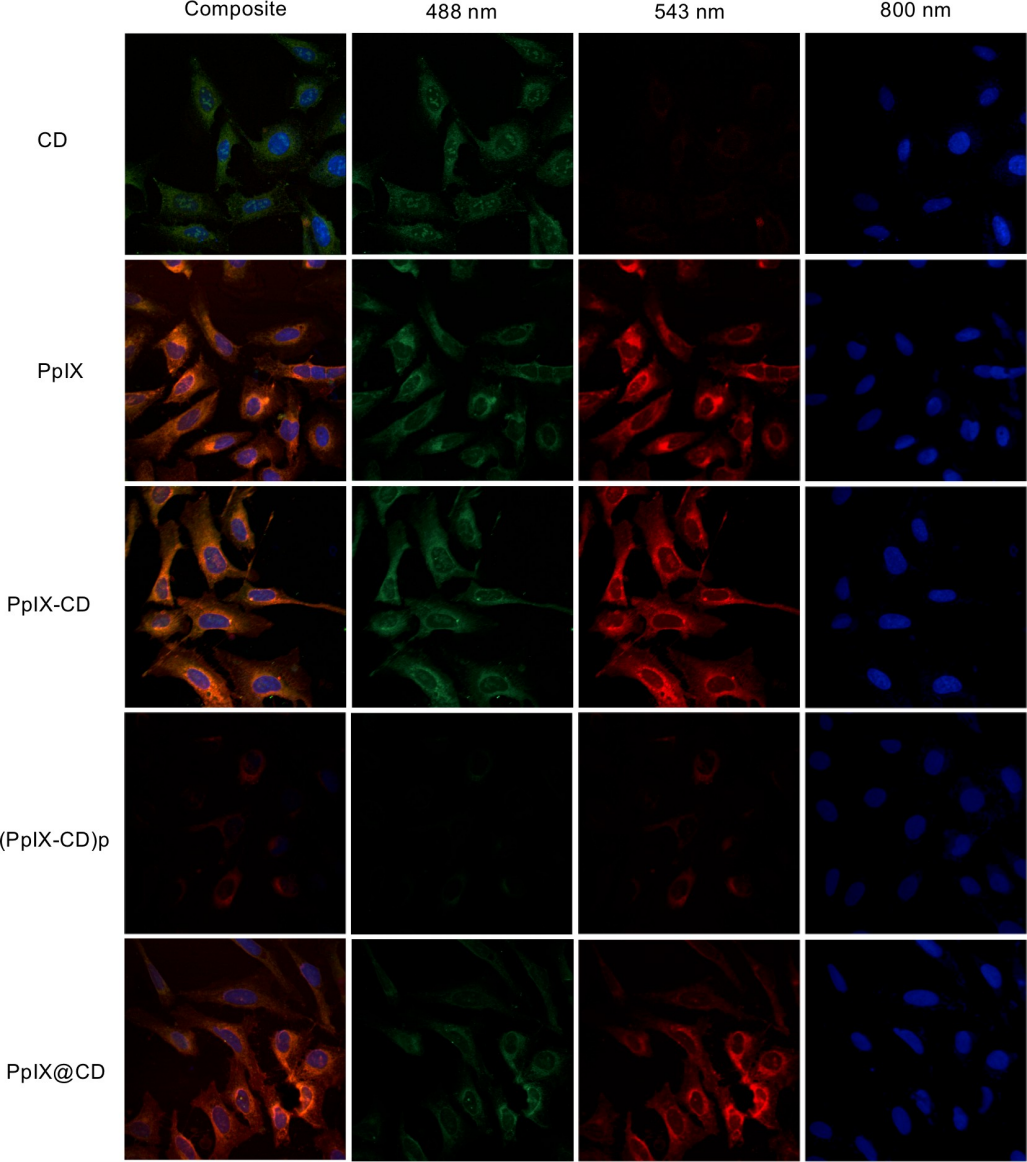
CD-PS conjugates can be used as probes for fluorescence imaging. CSLM images of U2-OS osteosarcoma. PpIX-CD and PpIX@CD have similar emissions to both CDs and PpIX, while (PpIX-CD)p has greatly decreased fluorescence emission. Conjugates appear to aggregate near the nuclei.

Confocal laser scanning microscopy (CSLM) was used to observe PpIX-CD and PpIX@CD uptake and distribution in osteosarcoma cells (U2-OS) (Fig 6). CSLM images show all conjugates within cells distributed along the cytoplasm and cell nuclei. There are small differences between the samples; PpIX-CD appears to have slightly more aggregates in comparison to PpIX@CD. (PpIX-CD)p has a noticeably decreased fluorescence intensity in comparison to all other samples and has a noticeably decreased emission at 488 and 543 nm. Conjugate concentration appears to be higher near the nucleus for PpIX-CD and PpIX@CD. Z-stacks showed both CDs and conjugates were localized near the nuclei in comparison to PpIX, which has no specific location for accumulation. We observed rapid uptake of all conjugate samples at various concentrations and time points.

## Discussion

We selected microwave-assisted pyrolysis because of its adaptability, ease of use, and rapid reaction time. In comparison, other protocols like hydrothermal synthesis or combustion are more difficult to standardize. The reaction temperature (150°C) and time (5 minutes) has previously been shown to be adequate for rapid CD synthesis with citric acid as a molecular precursor.[40] PpIX has greater thermal stability in comparison to the other reagents used for CD synthesis (citric acid or sucrose, ethylenediamine). The majority of the molecular mass is based on the pyrrole ring, which begins thermal decomposition around 360–450°C as evidenced by TGA analysis. Although there is a slight mass reduction from 26–360°C, this has been mainly linked to the breaking of reactive bonds outside the ring [41]. Therefore, it is highly likely host-guest embedding does not significantly alter PpIX structure and functionality during microwave-assisted synthesis. Furthermore, the high thermal stability of the guest molecule (PpIX) at the synthesis temperature suggests no other CDs are formed aside from those obtained by sucrose and EDA pyrolysis.

We observed large ash-like aggregates formed during the carbonization of citric acid and sucrose. Therefore, we adjusted the initial carbon source concentration and continuously stirred the solution throughout the process. The presence of precipitate in embedded carbon dots during microwave synthesis has been previously reported [36]. Although this fraction can be removed through centrifugation or ultracentrifugation, a large quantity of the dopant is lost in this step. We have observed that the use of a microwave reactor with stirring increases the total quantity of PpIX that could be introduced efficiently with host-guest embedding is around 1 wt%. In comparison, previously reported embedded Nile Blue in PEG-based CDs used a 1:10 weight ratio and demonstrated a higher degree of aggregate formation, requiring centrifugation at 20,000 g and the use of an aqueous gel separation column to recover the sample [42]. Dialysis removed remaining compounds from synthesis and cross-linking such as polycyclic aromatic compounds, buffer salts, and unbound PpIX [43].

EDC-NHS cross-linking ensures the directional cross-linking of CDs to PpIX and avoid the formation of dimers. The standard protocol is that of protein cross-linking with an activation buffer for the component with carboxyl groups (0.1 M MES, 0.5 M sodium chloride in deionised water) at pH 6. However, PpIX has very low solubility in water and readily aggregated when at low concentrations (<50 μg/ml). Yildiz *et al*. (2010) fabricated a similar conjugate using a 50:50 DMSO/water solution to improve compound solubility prior to cross-linking [44]. We experimented with water-miscible solvents for PpIX including acetone, DMSO, and DMF. We determined DMF to be the most suitable solvent for cross-linking after several trials.

TEM images demonstrate a size and morphology variation between conjugates. We speculate this is caused by the synthesis strategy and influences particle solubility. PpIX-CD was fabricated in a controlled and directed cross-linking reaction utilising purified CA-EDA CDs and PpIX. We also observed (PpIX-CD)p is the most heterogeneous sample with a wide size distribution. Host-guest embedding relied on the one-pot synthesis of PpIX@CD which produced slightly larger aggregates. CD nucleation and growth is altered by various synthesis conditions, such as temperature and type of carbon source. This could be further affected by the use of hydrophobic compounds and may have led to greater size variation in PpIX@CD compared to PpIX-CD [45]. Therefore, host-guest embedding appears to be a less reliable and reproducible strategy for drug loading in CDs in comparison to amide cross-linking.

A dual emission behaviour was observed in all conjugates with intense and broad emissions at the 420–520 nm range similar to CD samples in literature [46]. Drug loading through both amide cross-linking and embedding directly reduced CD fluorescence. This reduction is likely caused by quenching of CD-based emissions through conjugate aggregation and obstruction of surface defects, which have been reported to heavily contribute to CD photoluminescence [47]. However, porphyrin-associated peaks to not seem to be greatly affected by either embedding or amide cross-linking at its emission peaks at 617 and 677 nm. This is possibly due to its outer location in the conjugate as PpIX binds to each CD. CDs typically have a strong and broad absorption in the ultraviolet region, followed by a constant decrease as excitation wavelength increases [48]. Absorption below 375 nm has been attributed to π–π* transitions of the carbon dot surface, mainly from C = C and C = N bonds [49]. Carbon dot conjugates showed great similarity to PpIX with an absorption peak evident at 405 nm. PpIX loading was calculated as stated in the literature by comparing the relative intensities of the 658 nm peak while exciting the solution at λ_ex_ = 404 nm [38]. The CD fluorescence was subtracted from each conjugate to estimate drug loading.

We divided conjugates as previously mentioned in two groups based on their similarities to CDs or PpIX. The reduction of available amine functional groups during amide cross-linking is likely the cause for variation between spectra. These slight variations between samples can be observed particularly in the distinctive amide peak at ~1570 cm^-1^. The change in the availability of primary amines can also be seen in the region of 918–625 cm^-1^ which has been previously linked to N-H wag in carbon dots [50]. The assignment of peaks was carried out by comparing IR spectra to those found in the literature for CD samples (S3 Table).

XPS analysis demonstrated there is little to no variation in the carbon envelopes of conjugates and PpIX. C = C bonds seen in the high resolution C1s spectra could present π-π* transitions, which could lead to small intensities at higher binding energies but should not be considered true XPS peaks. We observed that the C-N environment is the major component and is seen at a higher binding energy and slightly reduced peak positions. This is possibly due to the influence of carbons attached to nitrogen in PpIX and conjugates as it contains a porphine core with a tetrapyrrole macrocycle, giving it an aromatic nature [51].

Singlet oxygen (O^1^_2_) production alone initially indicates (PpIX-CD)p is the best conjugate for PDT, while PpIX-CD and PpIX@CD appear to have reduced efficiency. However, this is because DMF was required for the measurement. O^1^_2_ yield was likely affected by the increased solubility of (PpIX-CD)p in organic solvents, as this conjugate readily aggregates in water. Nonetheless, we observed all the samples showed decreased singlet oxygen emission in comparison to PpIX. Our results indicate fluorescence emission intensity of the conjugates cannot be directly linked to singlet oxygen yield.

CDs displayed extremely low toxicity with LD_50_ values (μg/ml) similar to previously reported particles [52]. CA-EDA and S-EDA showed over 75% metabolic activity at concentrations up to 250 μg/ml. In comparison, all PpIX-containing samples showed a sharp reduction in metabolic activity over 50 μg/ml. Dark toxicity values of conjugates uniformly showed statistical difference from PpIX from concentrations ≥5 μg/ml (*p* < 0.05). The change from μg/ml to PpIX-adjusted concentrations showed samples are capable of delivering higher quantities of PpIX without affecting the cells regardless of loading strategy. We speculate conjugate toxicity may be influenced by solubility in an aqueous environment, incubation time, and cell type. PpIX is a highly hydrophobic compound with approximately 1 μg/ml solubility in water [53]. The decrease in dark toxicity of CD-based conjugates is likely a combination of more innocuous intracellular localization and decreased formation of aggregates after uptake due to the presence of cross-linked nanoparticles.

We observed light-activated toxicity in all samples. Additionally, we determined singlet oxygen yield did is not directly linked to increased phototoxicity for CD conjugates. The control PpIX showed no significant difference from PpIX-CD and PpIX@CD throughout various time points and concentrations >1 μg/ml. Nonetheless, both samples showed increased efficiency as an equivalent phototoxic effect was achieved utilising 36–42% loaded PpIX. We observed a 3.2 to 4.1-fold increase in photo-toxicity index (PI) compared to PpIX. The precipitate fraction (PpIX-CD)p had previously shown a high singlet oxygen yield in DMF but failed to produce a significant phototoxic effect in comparison to the control. It showed a reduced efficiency compared to other PpIX-conjugates with a LD_50_ of 7.2 μg/ml (4.3 μM PpIX). (PpIX-CD)p also showed a high degree of variation during light activation, which reduces its value as a photosensitizer. Its diminished water solubility in comparison to PpIX-CD may be caused by the formation of multiple covalent bonds during cross-linking leading to self-quenching.

Fowley et al. (2013) first reported the formation of a PpIX and CD conjugate with a Förster resonance energy transfer (FRET) mechanism for enhanced PDT. In this study,the decreased dark toxicity based on the increase of PpIX solubility was also observed with HeLa cells. Furthermore, single and two-photon activation of the conjugates was reported [35]. Therefore, it is possible an enhanced PDT effect could be produced by PpIX-CD and PpIX@CD due to the same FRET mechanism using a lower wavelength for CD excitation or via two-photon irradiation. Although the two-photon activation of conjugates is limited by the reduced area, two-photon PDT is considered to be a highly promising future trend for research. This modality is capable of a selective and highly targeted treatment for a wide variety of conditions such as brain tumours, atherosclerosis, and other deep-seated cancers, while limiting damage to surrounding healthy tissue. Although two-photon photosensitisers have been successfully fabricated and evaluated *in vitro*, a considerable improvement is expected through the use of vectors such as CDs for more efficient intracellular localisation.

The intracellular localization of conjugates may also benefit the action mechanism of singlet oxygen. The intracellular accumulation of PpIX in mitochondria has been previously reported based on its uptake by binding to a mitochondrial translocator protein involved in the heme biosynthesis pathway [54]. Additionally, porphyrins have been shown to inhibit several important mitochondrial enzymes leading to the inhibition of oxidative phosphorylation [55]. PpIX-based conjugates appear to follow these previously described interactions, with the advantage of slightly increased water solubility due to the presence of CDs. However, the mechanism of cell death after photoactivation of CD-PpIX conjugates is still unclear. PpIX-induced cell death has been shown to be p53-dependent and independent; Zawacka-Pankau et al (2006) proposed PpIX sensitizes cancer cells making them susceptible to PDT and disrupting proliferation through the destabilization of the HDM2-p53 complex in the mitochondria [56]. Our results suggest that the variation of incubation time before light exposure may also influence phototoxicity.

Hua et al. (2018) reported a similar conjugate based on the cross-linking of CDs and PpIX with dicyclohexylcarbodiimide and 1-hydroxybenzotriazole (DCC/HOBt). They reported increased cellular uptake and decreased toxicity prior to photoactivation. Interestingly, these conjugates presented a similar size (25.2 ± 5.7 nm) to that of our cross-linked conjugate (25 ± 10 nm), but lower PpIX loading efficiency of 23.3%, in comparison to 43.3% (PpIX-CD) and 35.59% (PpIX@CD). Furthermore, they reported an intrinsic nucleolus-targeting capability better than the only commercially-available dye SYTO RNASelect [57]. Although DCC has some drawbacks such as the need for an organic solvent during cross-linking, it benefits from the lack of hydrolysis during the reaction and low cost compared to EDC. DCC/HOBt cross-linking has been used previously with CDs to conjugate photosensitizer Rose Bengal and the mitochondria targeting moiety triphenylphosphonium [58,59]. Similarly, Zheng et al. (2016) demonstrated efficient PDT with a carbon nitride (C_3_N_4_)-based multifunctional nanocomposite (PCCN) consisting of CDs, Arg-Gly-Asp motif, and PpIX. PCCN demonstrated water-splitting ability to produce singlet oxygen production while in a state of hypoxia with a PpIX content of 9.6%. [60] These studies further demonstrate the versatility of CDs as parts of hybrid systems for efficient PDT.

CSLM images demonstrate PpIX, PpIX-CD and PpIX@CD are similar as bioimaging probes and are mostly distributed along the cytoplasm, with strong emission at 543 nm excitation. PpIX@CD demonstrates slightly reduced photoluminescence at 488 nm in comparison to PpIX and PpIX-CD. Confocal z-stacks clearly show conjugates and PpIX do not enter the nucleus but instead accumulate around the centre of the cell. Cancer cells have been shown to have increased mitochondria in the perinuclear area, which is consistent with our observations [61]. In comparison, CDs (CA-EDA and S-EDA) do not show significant fluorescence after excitation at 543 nm but show some aggregates within the nucleus. We speculate this is due to their decreased size and faster uptake in comparison to conjugates. Finally, (PpIX-CD)p shows greatly reduced fluorescence emission with the 488 and 543 nm laser lines. This signal reduction could be caused by multiple factors. We have previously observed decreased fluorescence intensity and rapid aggregation of (PpIX-CD)p, which has been shown to cause quenching as carbon dots [62] and PpIX [12]. Our results indicate PpIX-CD and PpIX@CD are capable of acting as high-contrast imaging probes without decreasing therapeutic efficiency for theranostics applications.

## Conclusion

PpIX was successfully encapsulated in carbon dots through one-pot microwave-assisted synthesis, PpIX@CD. PPIX-CD and (PpIX-CD)p were synthesized through carbodiimide cross-linking of CDs and PpIX. Host-guest embedding was shown to be a viable and cost-effective alternative to carbodiimide cross-linking for loading PpIX and increasing photo-index. Nonetheless, sample variability requires the optimization of synthesis conditions. PpIX loading in carbon dot conjugates reduced CD-attributed photoluminescence and singlet oxygen generation in all conjugates regardless of drug loading percentage. Our results demonstrate host-guest encapsulated PpIX@CD and carbodiimide-linked PpIX-CD conjugates produce similar PDT effect to that of PpIX with a lower drug concentration, increasing the therapeutic window of the compound. We expect CD-based conjugates to have significant value in biomedical applications as carriers in PDT and PDD, as well as biomedical applications related to theragnostics, drug delivery, and bioimaging.

## Supporting information

S1 Fig (PpIX-CD)p in water. TEM images of (PpIX-CD)p at 18,500X (bottom). Individual particles can be observed around the edges of the aggregate.
(TIFF)

S2 Fig CDs in water. TEM images of CDs at 68,000X (bottom). S-EDA and CA-EDA CDs both show quasispherical morphology.
(TIFF)

S3 Fig FT-IR spectra. FT-IR spectra of conjugated samples in the range of 4000–700 cm^-1^.
(TIFF)

S4 Fig UV-Vis absorption. Absorbance spectra of PpIX, PpIX-CD, (PpIX-CD)p and PpIX@CD.
(TIFF)

S5 Fig XPS C 1s scan of PpIX. (TIFF)

S6 Fig XPS survey scan of PpIX. (TIFF)

S7 Fig XPS C 1s scan of PpIX-CD. (TIFF)

S8 Fig XPS survey scan of PpIX-CD. (TIFF)

S9 Fig XPS C 1s scan of (PpIX-CD)p. (TIFF)

S10 Fig XPS survey scan of (PpIX-CD)p. (TIFF)

S11 Fig XPS C 1s scan of PpIX@CD. (TIFF)

S12 Fig XPS survey scan of PpIX@CD. (TIFF)

S1 Table High resolution C1s spectra. Curve fitting of the C 1S high resolution spectra of PpIX and CD-conjugates.
(TIFF)

S2 Table XPS of indium foil. Surface composition (atomic%) of indium foil.
(TIFF)

S3 Table FT-IR peak assignation. Table with assigned FT-IR peaks in conjugates in the range of 2000–700 cm^-1^.
(TIFF)

S4 Table Data—Dark toxicity of CDs and conjugates. (TIFF)

S5 Table Data—Light-activated toxicity (24 hours). (TIFF)

S6 Table Data—Light-activated toxicity (48 hours). (TIFF)

S7 Table Data—Light-activated toxicity (72 hours). (TIFF)
