# Supplementary figures and images for: Carbon dot-protoporphyrin IX conjugates for improved drug delivery and bioimaging
Source: PLoS One. 2019 Jul 25;14(7):e0220210. doi: 10.1371/journal.pone.0220210 (PMC6657888; doi:10.1371/journal.pone.0220210)

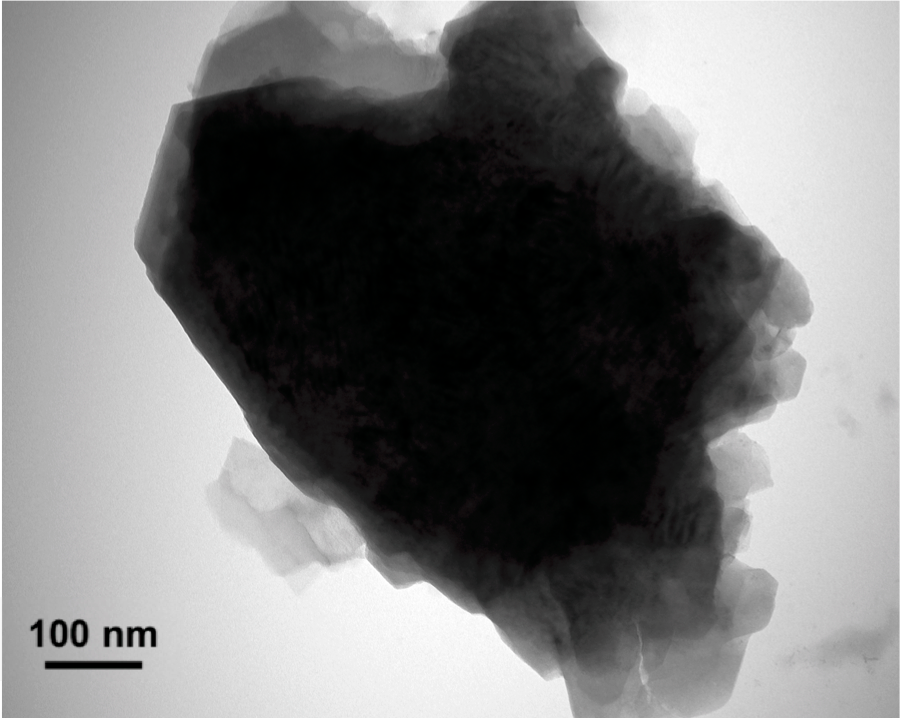

Supplement: S1 Fig — TEM images of (PpIX-CD)p at 18,500X (bottom). Individual particles can be observed around the edges of the aggregate. (TIFF) [file pone.0220210.s001.tiff]

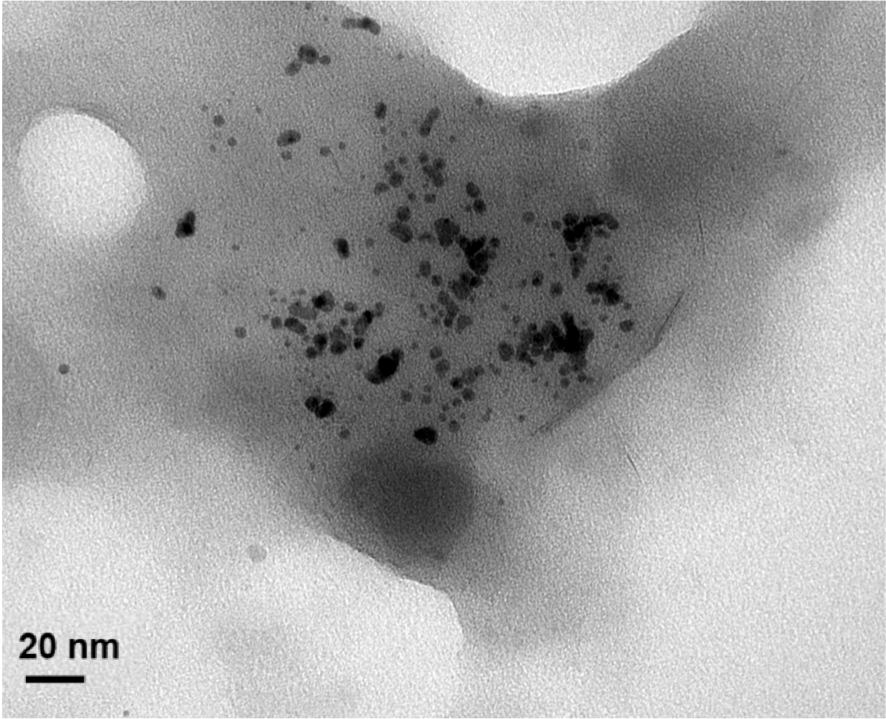

Supplement: S2 Fig — TEM images of CDs at 68,000X (bottom). S-EDA and CA-EDA CDs both show quasispherical morphology. (TIFF) [file pone.0220210.s002.tiff]

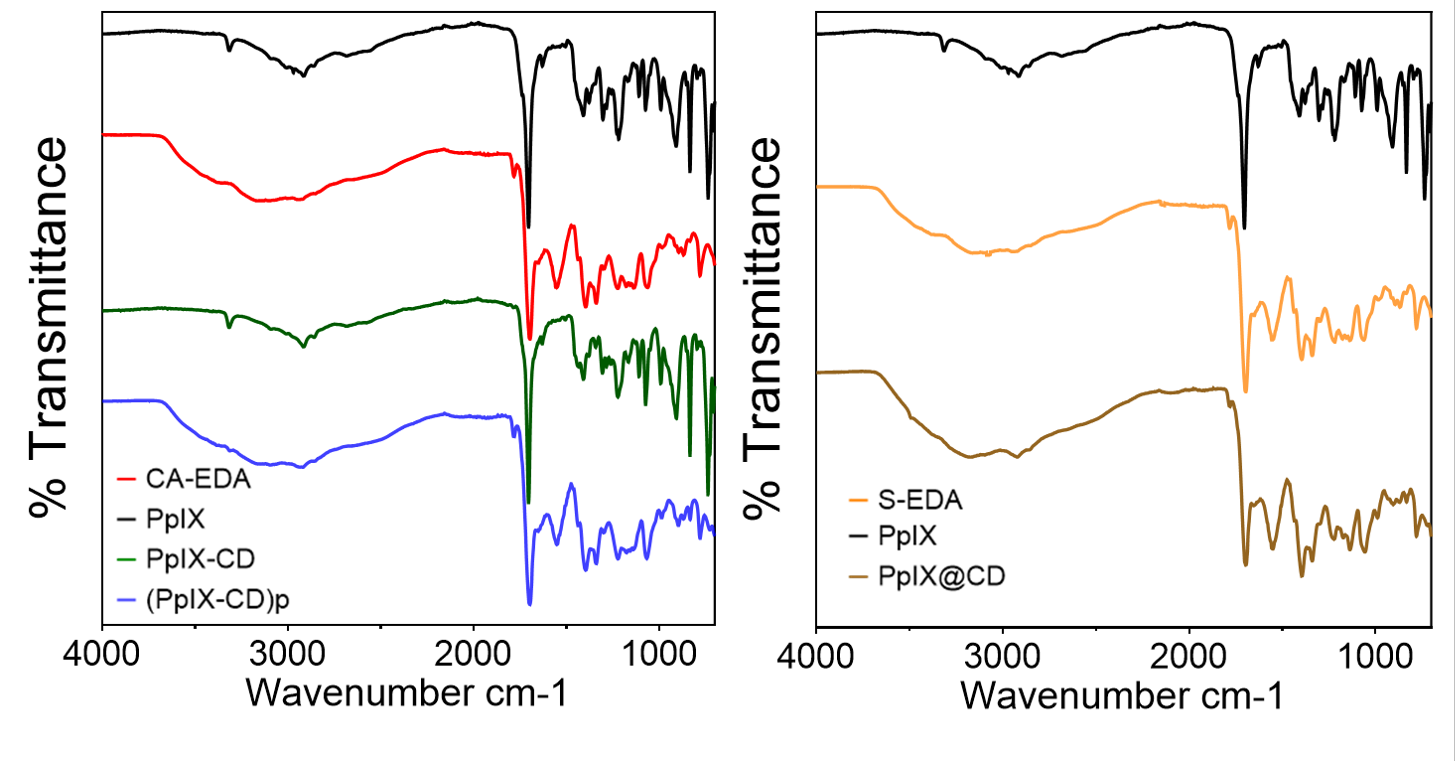

Supplement: S3 Fig — FT-IR spectra of conjugated samples in the range of 4000–700 cm-1. (TIFF) [file pone.0220210.s003.tiff]

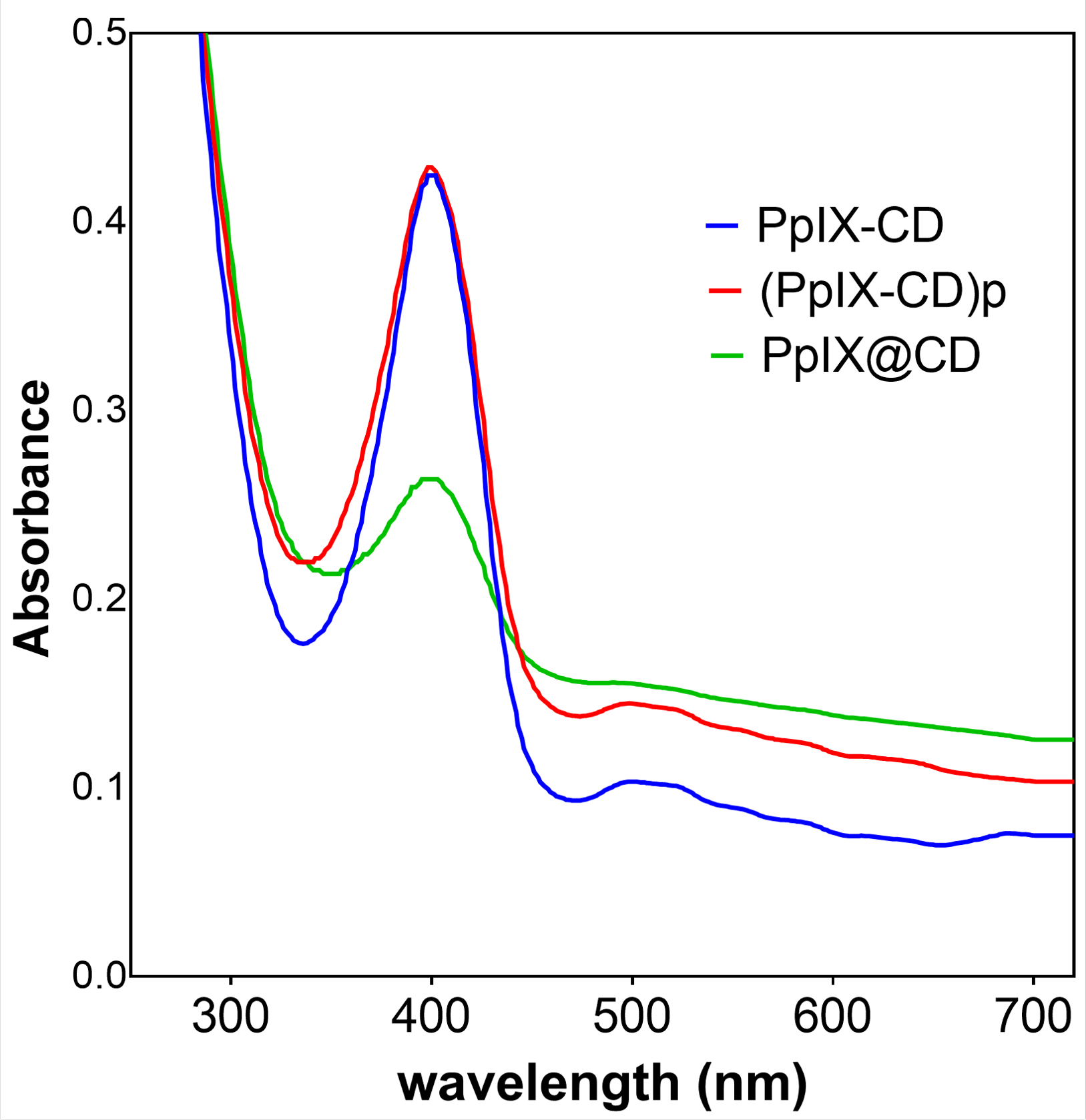

Supplement: S4 Fig — Absorbance spectra of PpIX, PpIX-CD, (PpIX-CD)p and PpIX@CD. (TIFF) [file pone.0220210.s004.tiff]

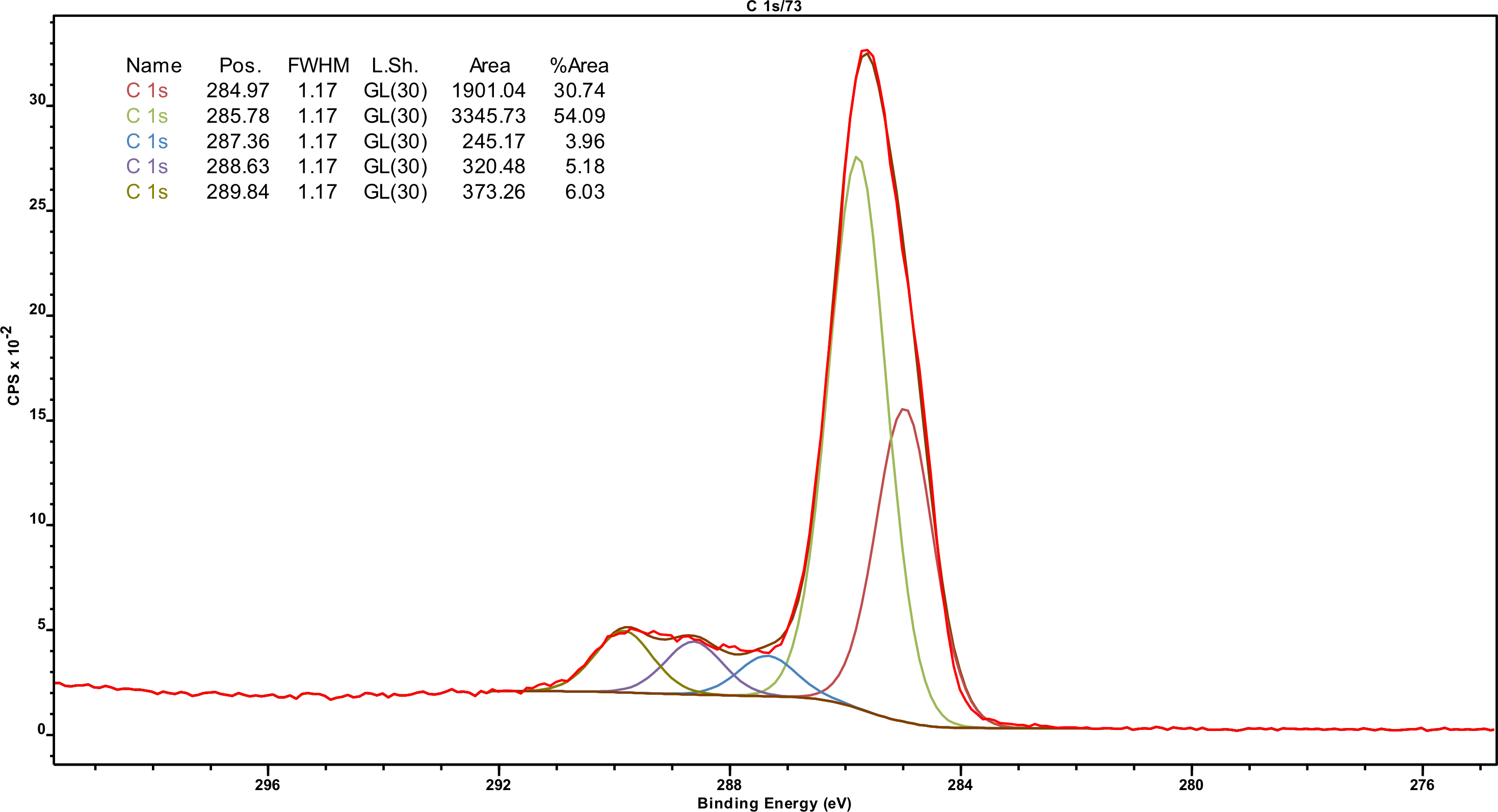

Supplement: S5 Fig — (TIFF) [file pone.0220210.s005.tiff]

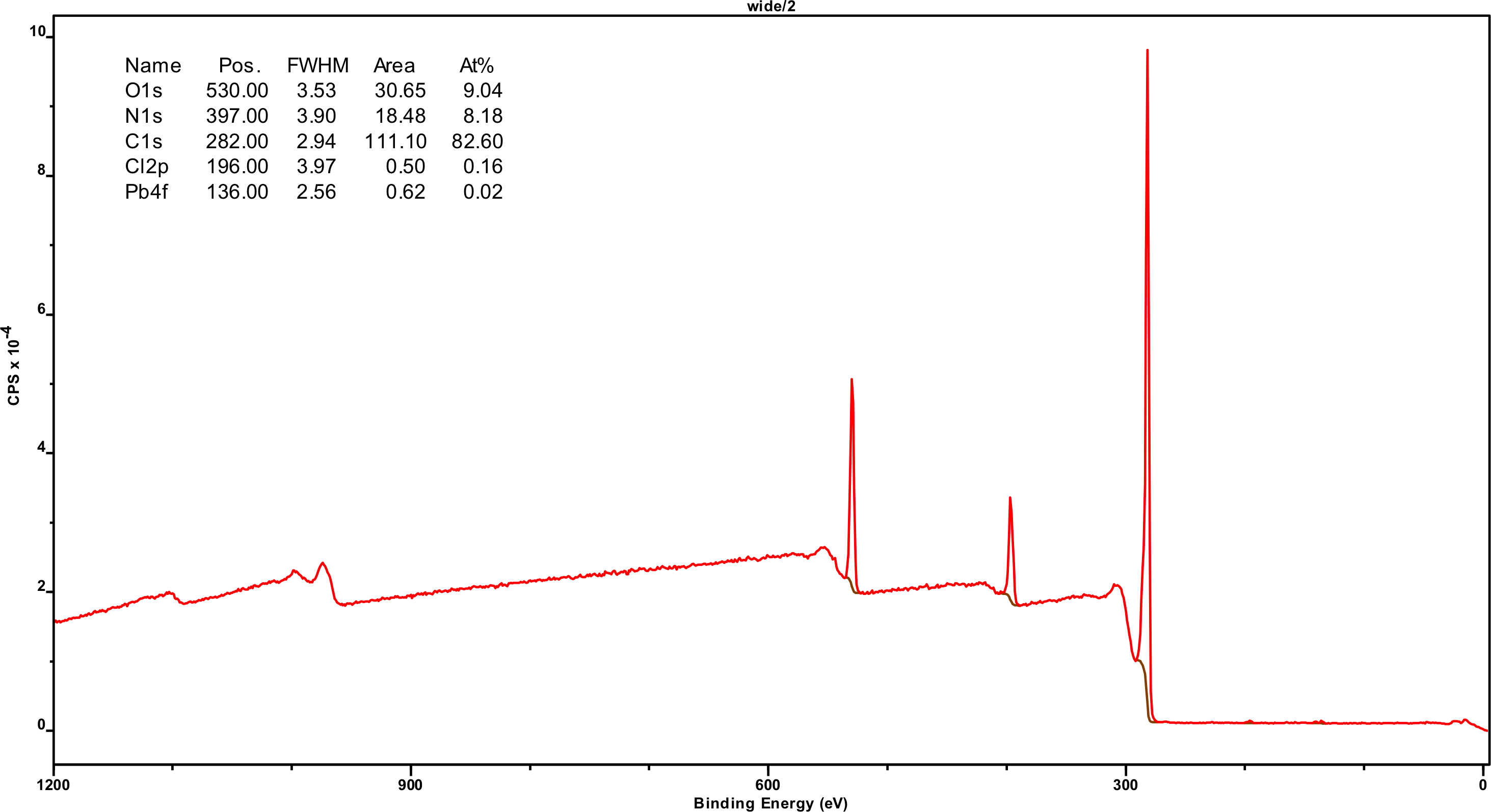

Supplement: S6 Fig — (TIFF) [file pone.0220210.s006.tiff]

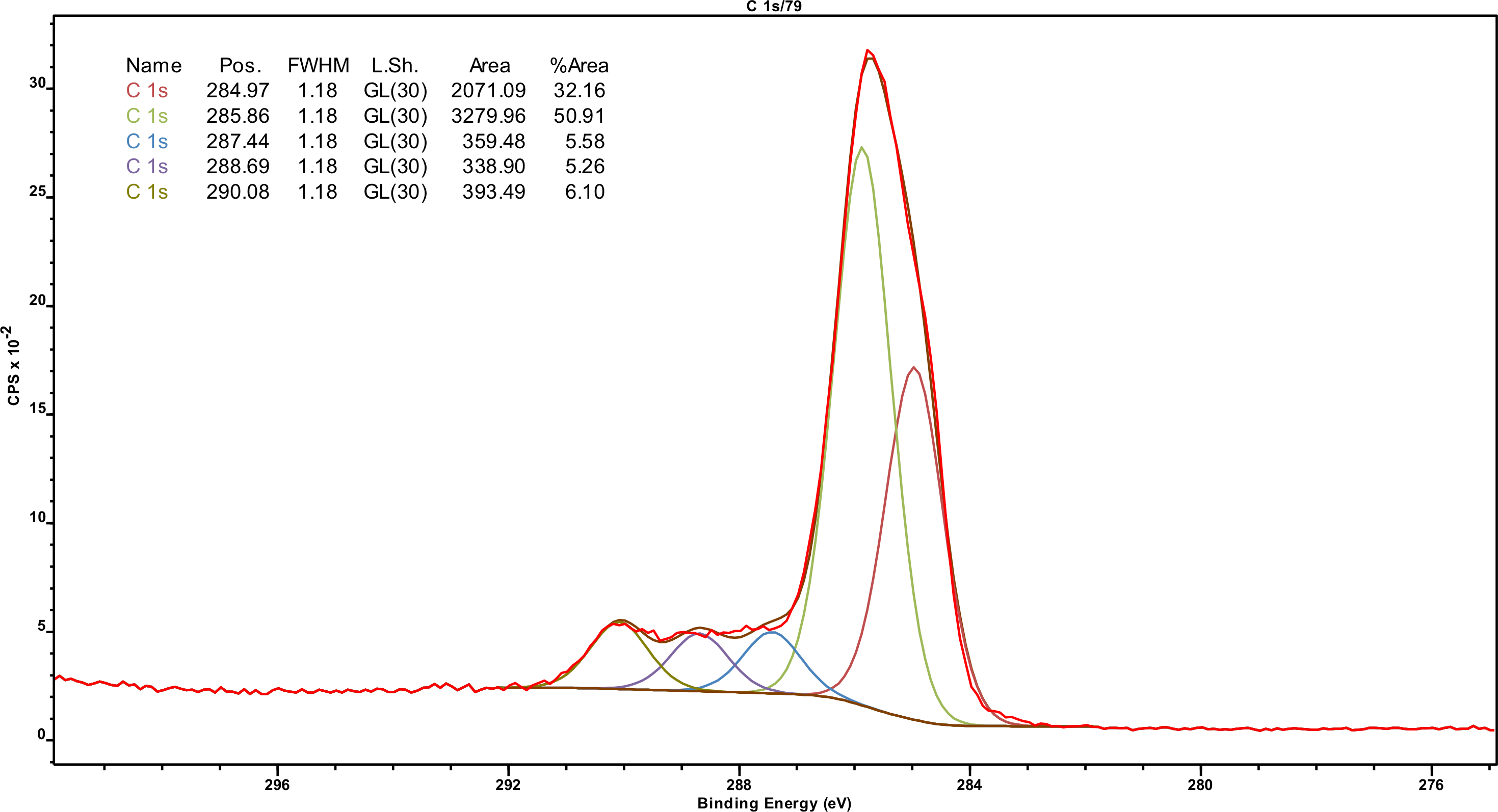

Supplement: S7 Fig — (TIFF) [file pone.0220210.s007.tiff]

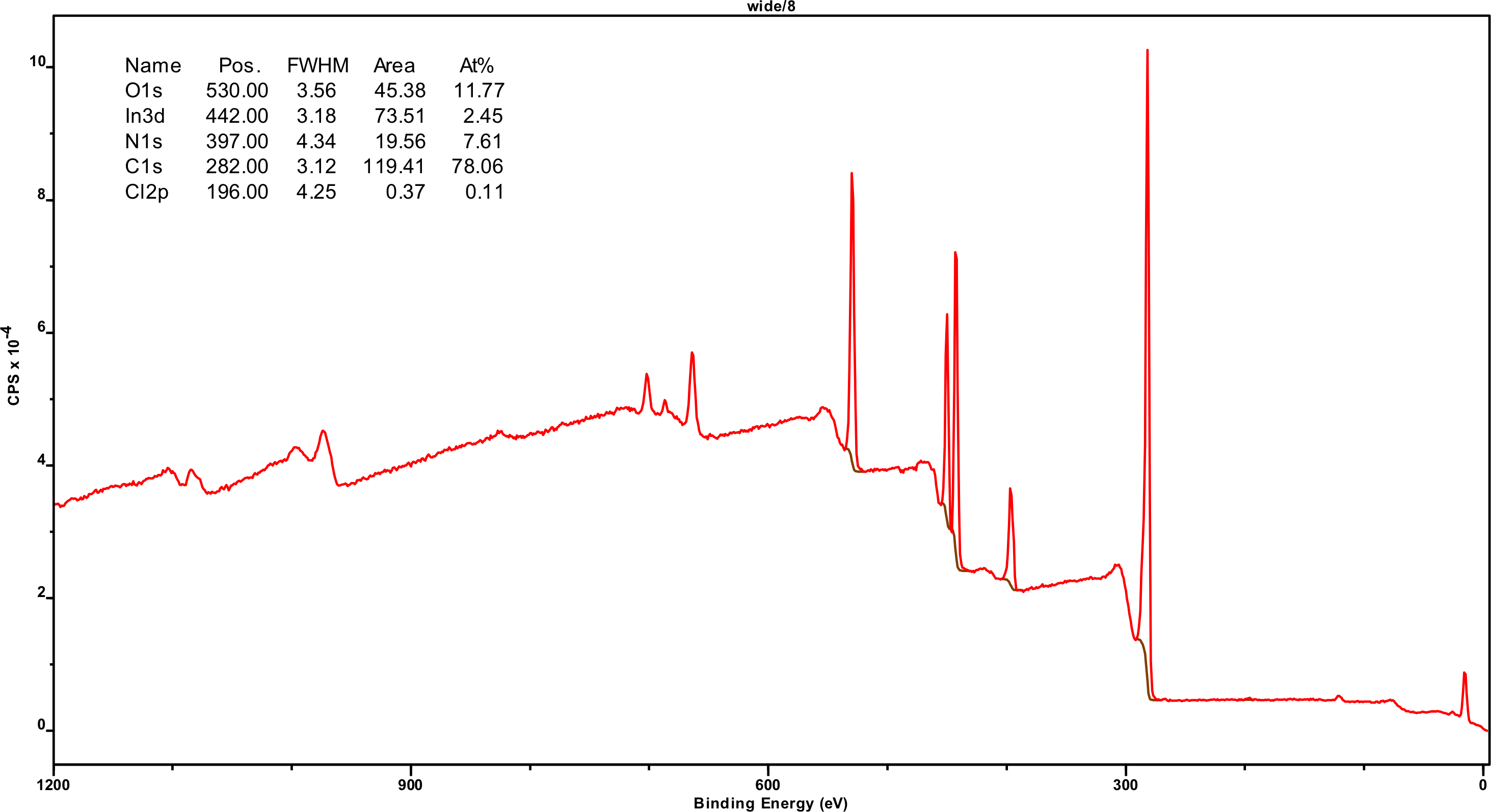

Supplement: S8 Fig — (TIFF) [file pone.0220210.s008.tiff]

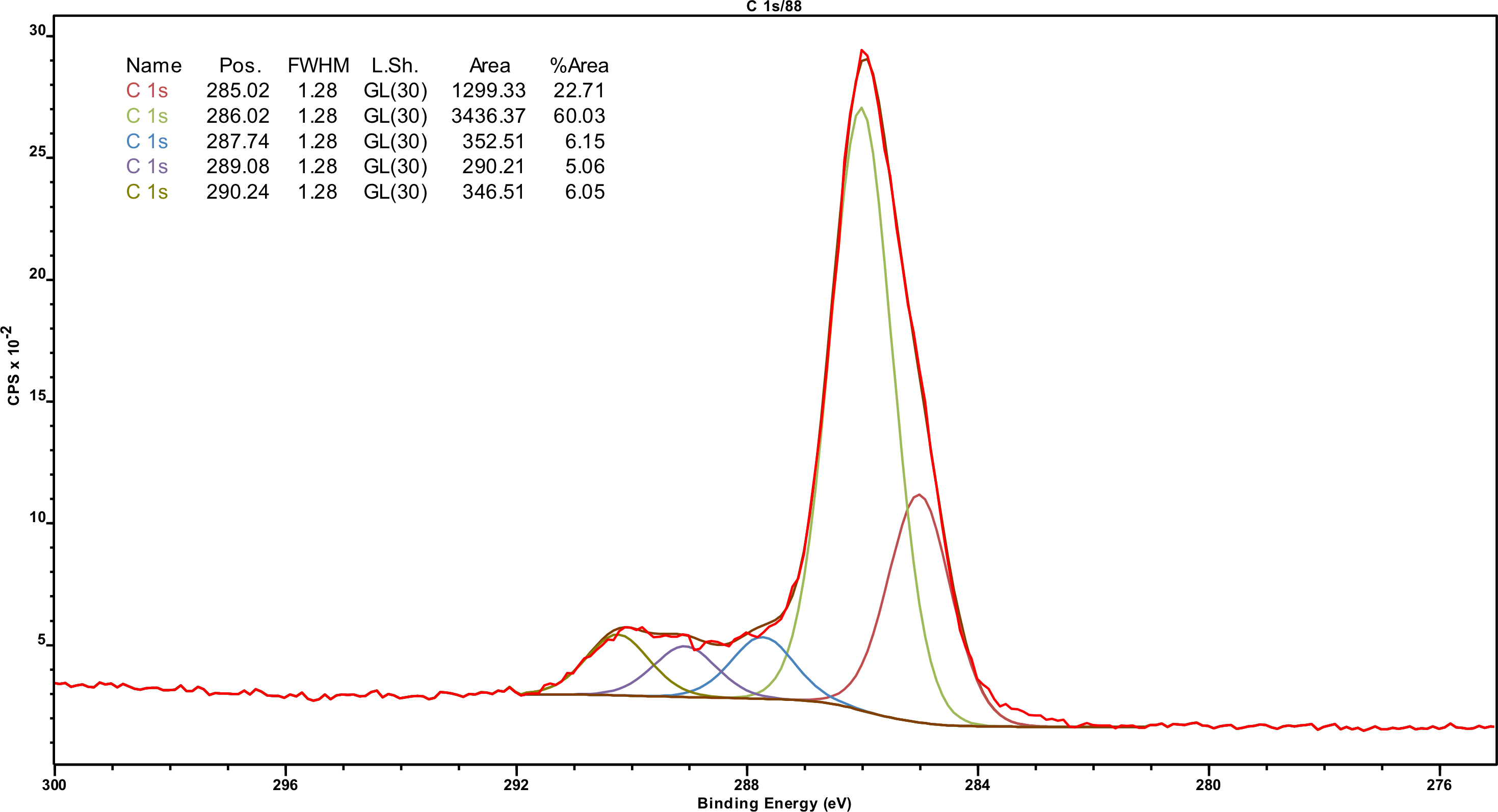

Supplement: S9 Fig — (TIFF) [file pone.0220210.s009.tiff]

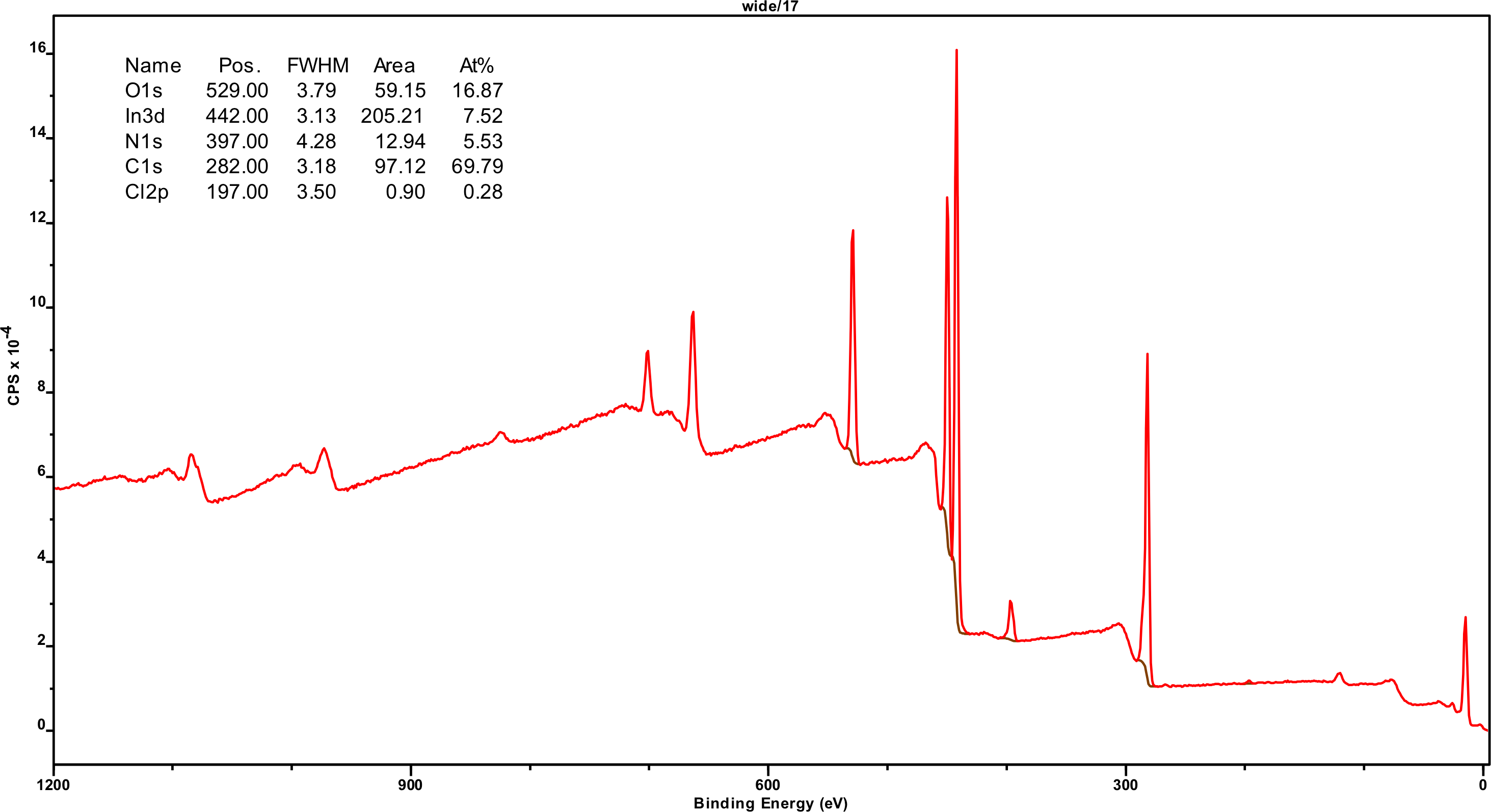

Supplement: S10 Fig — (TIFF) [file pone.0220210.s010.tiff]

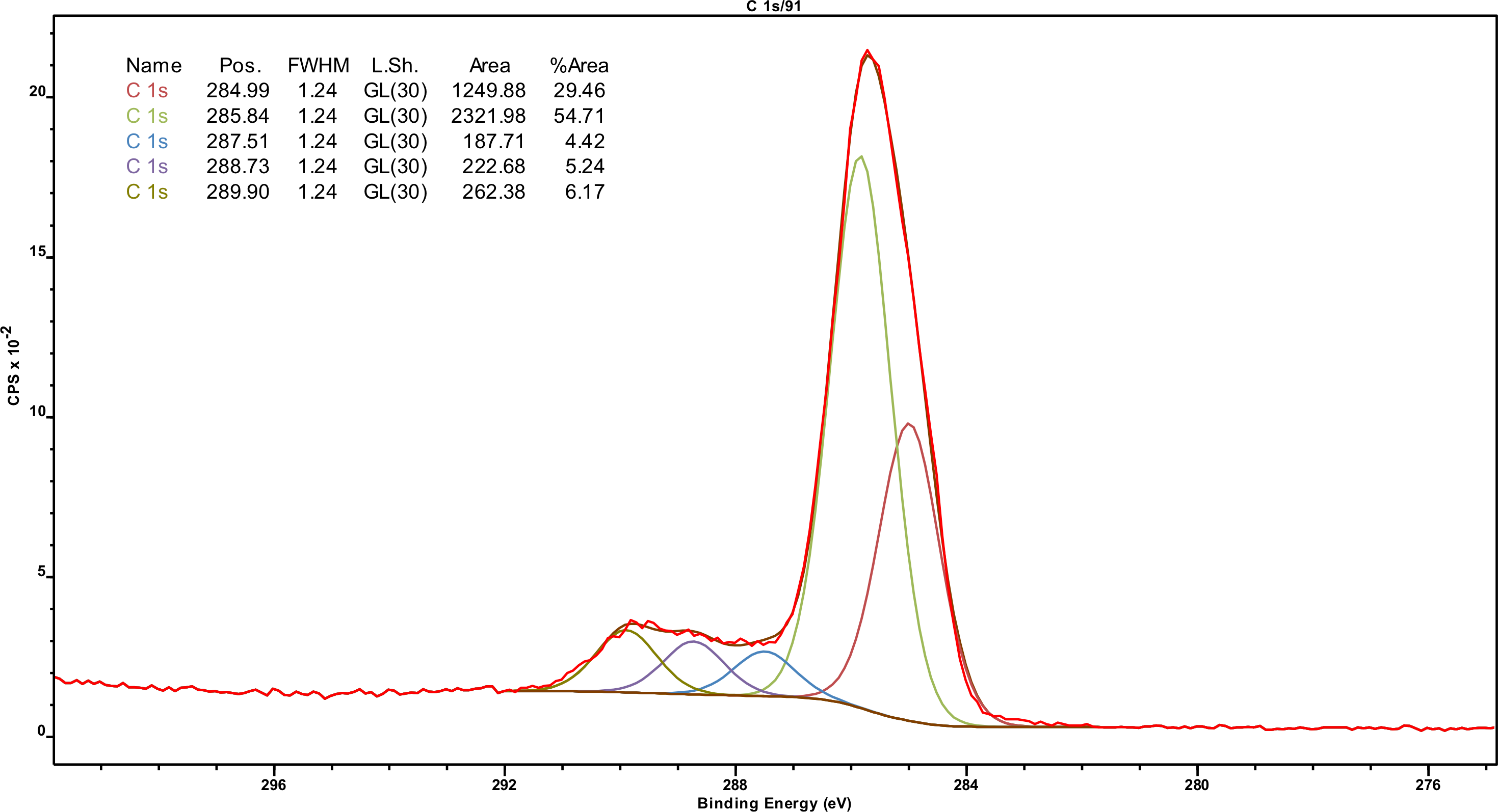

Supplement: S11 Fig — (TIFF) [file pone.0220210.s011.tiff]

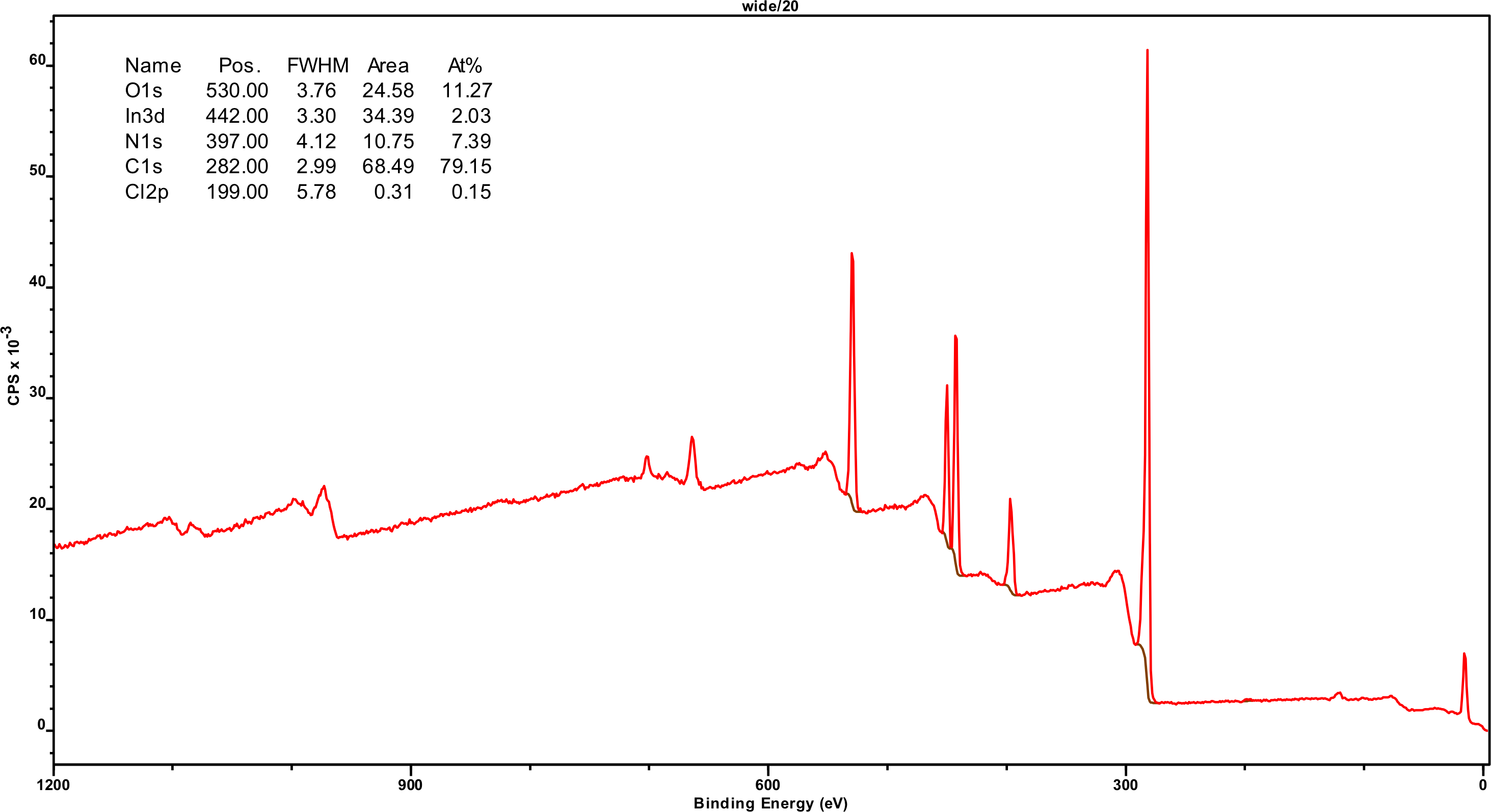

Supplement: S12 Fig — (TIFF) [file pone.0220210.s012.tiff]

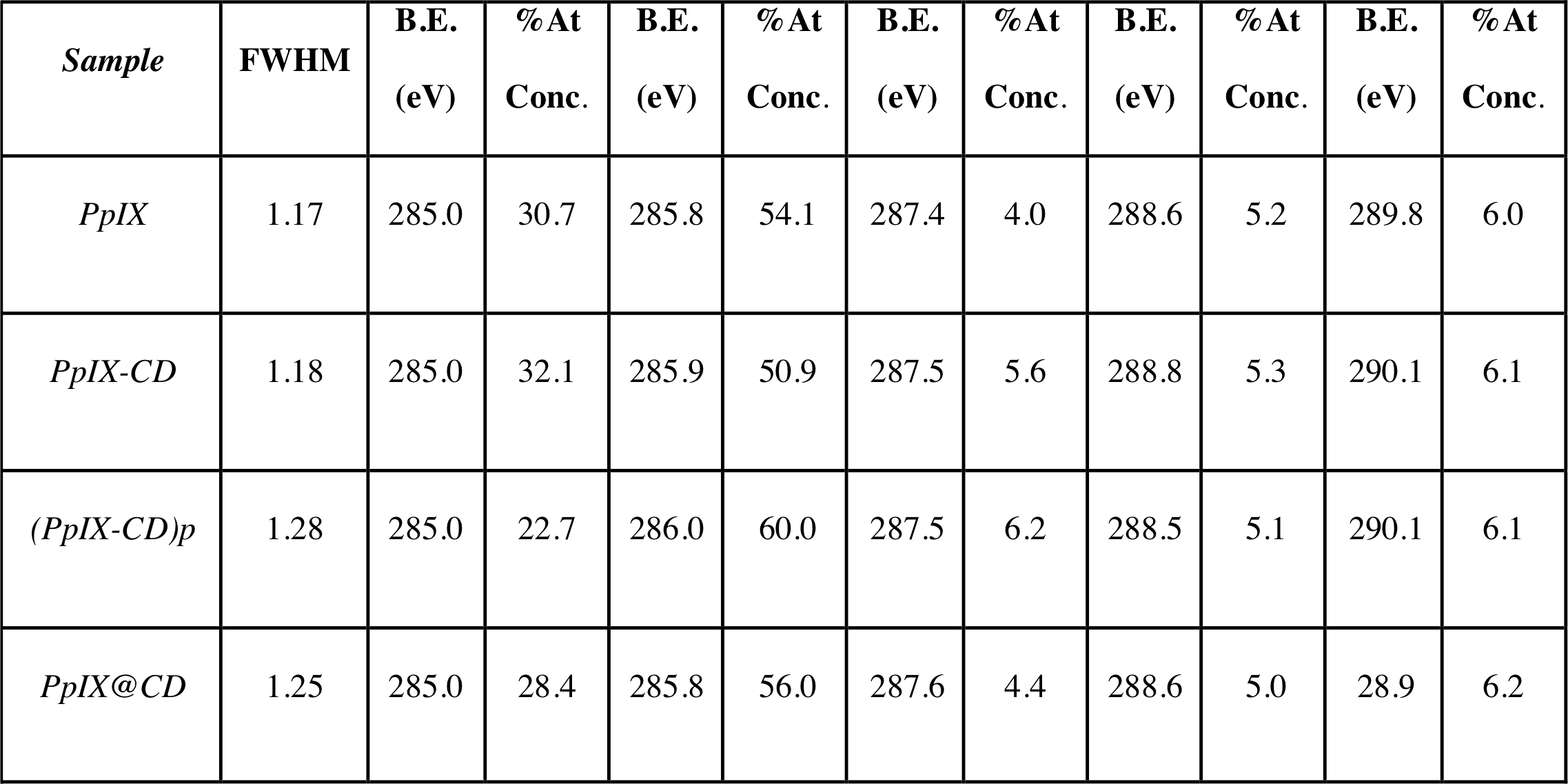

Supplement: S1 Table — Curve fitting of the C 1S high resolution spectra of PpIX and CD-conjugates. (TIFF) [file pone.0220210.s013.tiff]

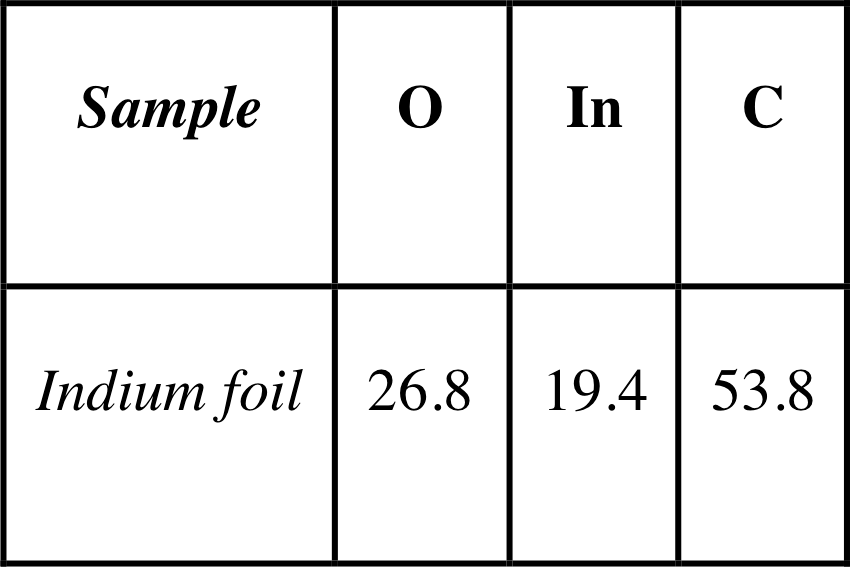

Supplement: S2 Table — Surface composition (atomic%) of indium foil. (TIFF) [file pone.0220210.s014.tiff]

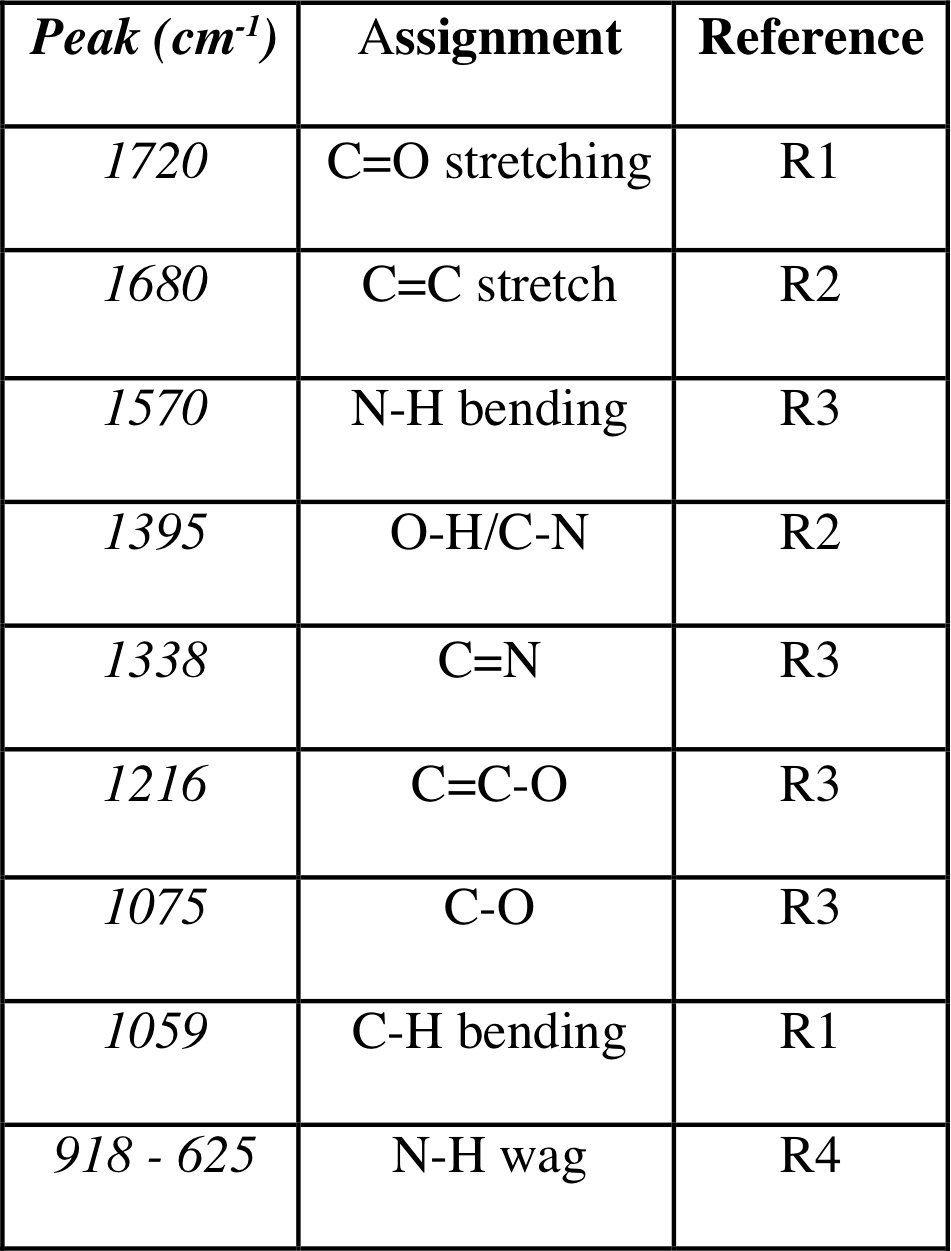

Supplement: S3 Table — Table with assigned FT-IR peaks in conjugates in the range of 2000–700 cm-1. (TIFF) [file pone.0220210.s015.tiff]

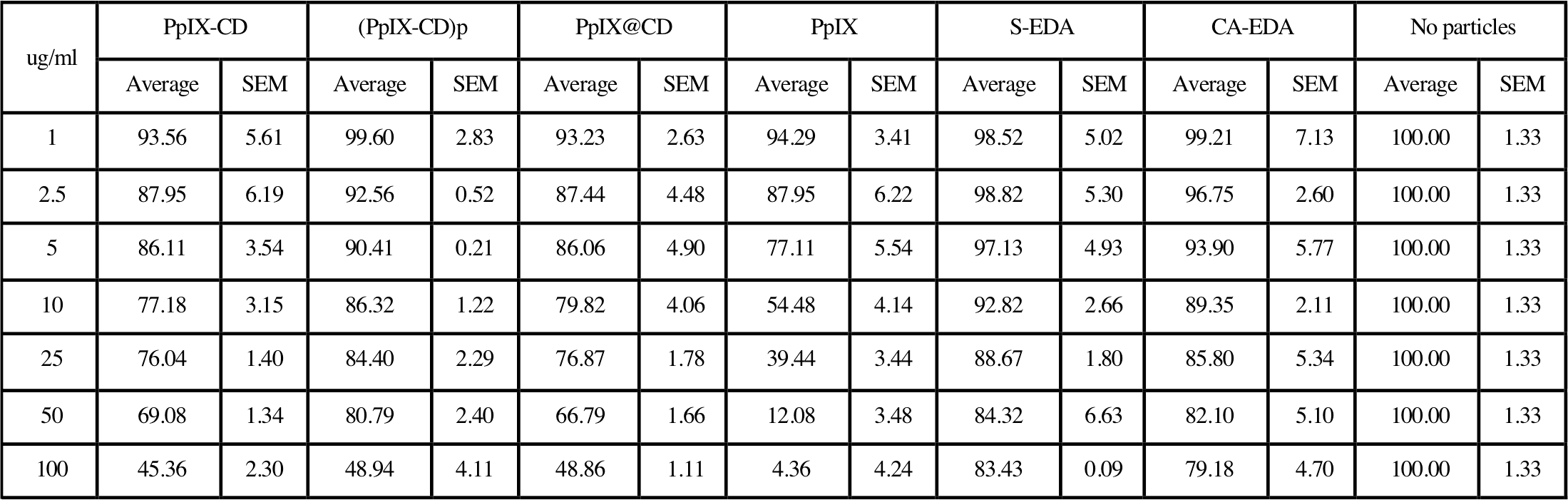

Supplement: S4 Table — (TIFF) [file pone.0220210.s016.tiff]

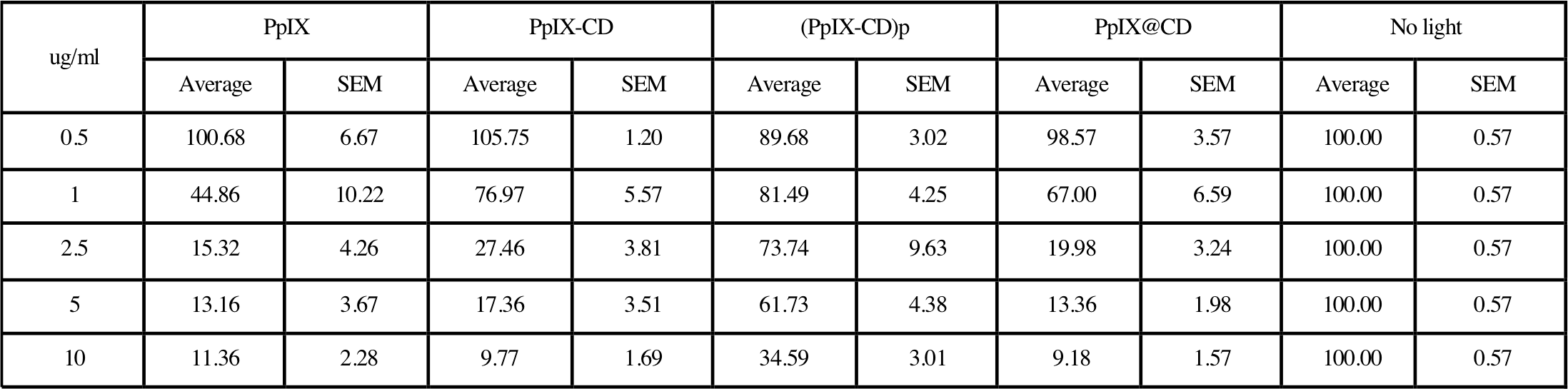

Supplement: S5 Table — (TIFF) [file pone.0220210.s017.tiff]

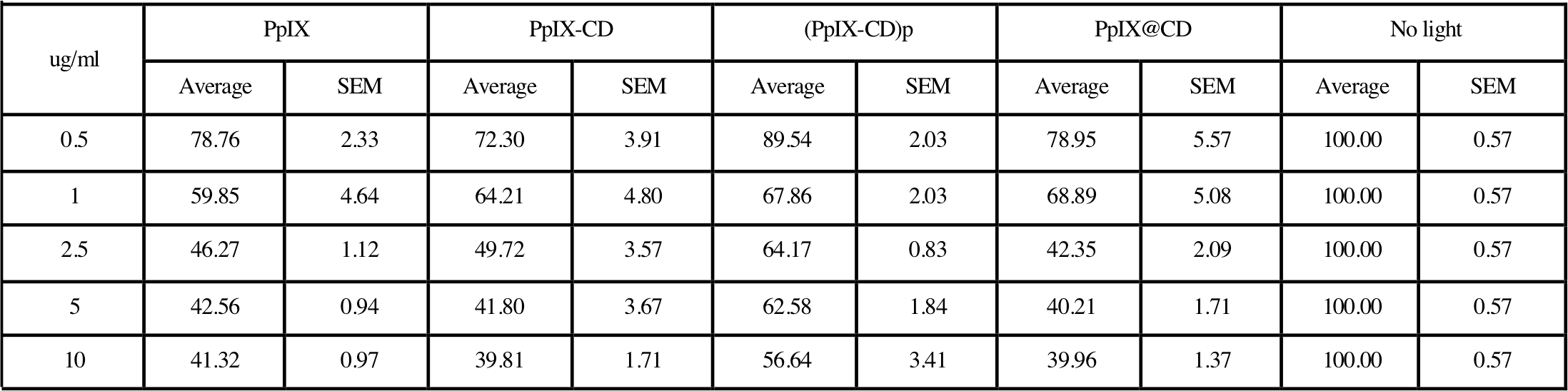

Supplement: S6 Table — (TIFF) [file pone.0220210.s018.tiff]

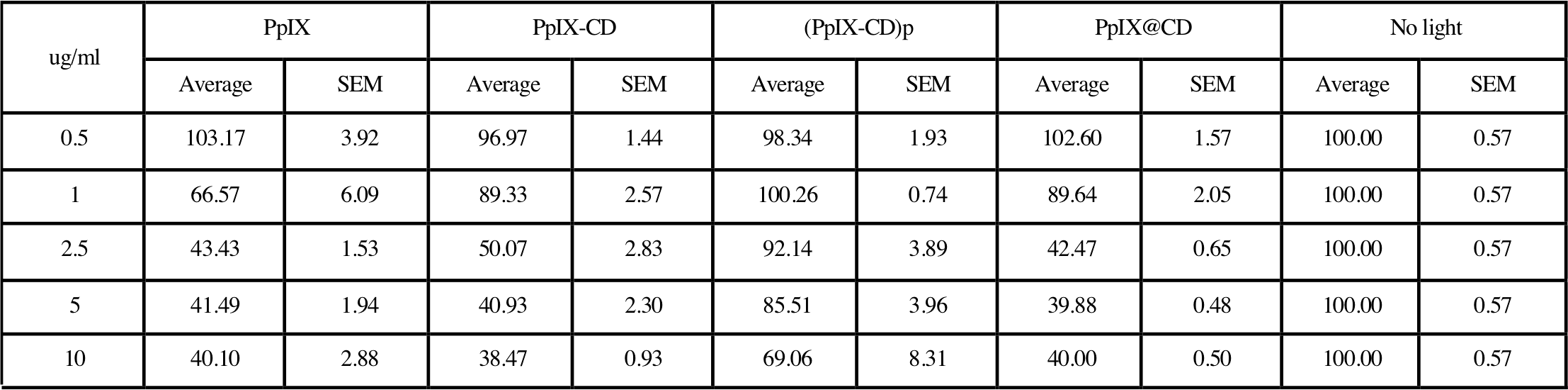

Supplement: S7 Table — (TIFF) [file pone.0220210.s019.tiff]
